# Low Temperature Affects Stem Cell Maintenance in *Brassica oleracea* Seedlings

**DOI:** 10.3389/fpls.2016.00800

**Published:** 2016-06-08

**Authors:** Jennifer de Jonge, Jan Kodde, Edouard I. Severing, Guusje Bonnema, Gerco C. Angenent, Richard G. H. Immink, Steven P. C. Groot

**Affiliations:** ^1^Bioscience, Plant Research International, Wageningen University and Research CenterWageningen, Netherlands; ^2^Laboratory of Molecular Biology, Wageningen University, Wageningen University and Research CenterWageningen, Netherlands; ^3^Wageningen UR Plant Breeding, Wageningen University, Wageningen University and Research CenterWageningen, Netherlands

**Keywords:** blind plants, *Brassica oleracea*, cell cycle activity, germination, seedlings, shoot apical meristem, stem cells

## Abstract

Most of the above ground tissues in higher plants originate from stem cells located in the shoot apical meristem (SAM). Several plant species can suffer from spontaneous stem cell arrest resulting in lack of further shoot development. In *Brassica oleracea* this SAM arrest is known as blindness and occurs in an unpredictable manner leading to considerable economic losses for plant raisers and farmers. Detailed analyses of seedlings showed that stem cell arrest is triggered by low temperatures during germination. To induce this arrest reproducibly and to study the effect of the environment, an assay was developed. The role of genetic variation on the susceptibility to develop blind seedlings was analyzed by a quantitative genetic mapping approach, using seeds from a double haploid population from a cross between broccoli and Chinese kale, produced at three locations. The analysis revealed, besides an effect of the seed production location, a region on linkage group C3 associated with blindness sensitivity. A subsequent dynamic genome-wide transcriptome analysis resulted in the identification of around 3000 differentially expressed genes early after blindness induction. A large number of cell cycle genes were en masse induced early during the development of blindness, whereas shortly after, all were down-regulated. This miss-regulation of core cell cycle genes is accompanied with a strong reduction of cells reaching the DNA replication phase. From the differentially expressed genes, 90 were located in the QTL region C3. Among them are two genes belonging to the MINICHROMOSOMAL MAINTENANCE gene family, known to be involved in DNA replication, a RETINOBLASTOMA-RELATED gene, a key regulator for cell cycle initiation, and several MutS homologs genes, involved in DNA repair. These genes are potential candidates for being involved in the development of blindness in *Brassica oleracea* sensitive genotypes.

## Introduction

Upon germination and during the vegetative stage of development, a small almost constant number of pluripotent stem cells is located in the shoot and root apical meristems (Yanai et al., 2005; van Zanten et al., 2011). The shoot apical meristem (SAM) can be divided into a central zone that is essential for maintenance of the meristem and a peripheral zone, from which lateral organs are initiated (Jürgens, 1995). The centrally localized stem cells are renewed throughout plant development, while a part of the cells in the SAM periphery differentiate to form the aerial parts of a plant (Barton and Poethig, 1993). In the plant model species *Arabidopsis thaliana*, maintenance of central stem cells is organized by a feedback loop between WUSCHEL, a homeobox transcription factor, and CLAVATA receptor-like kinases and ligand proteins (Laux et al., 1996; Schoof et al., 2000).

When the balance between maintenance of stem cells and cell differentiation is distorted, plants can lose SAM function. Loss of shoot development has been described for several plant species, such as tomato (*Solanum lycopersicum*; Wetzstein and Vavrina, 2002), baby’s breath (*Gypsophila paniculata*; Hicklenton et al., 1993), cauliflower and broccoli (both *Brassica oleracea;* Wiebosch et al., 1950). In general, unfavorable environmental conditions are believed to cause loss of SAM activity in sensitive plants. The plant environment varies regularly, ranging from optimal conditions for plant growth and development toward abiotic or biotic stress conditions (Janská et al., 2010). Plants have the ability to adapt to abiotic stress (e.g., temperature, drought, and salt) via specific signaling pathways involving phytohormones and regulatory proteins, such as receptors, protein modifiers, and transcription factors, ultimately leading to gene expression changes (Koornneef et al., 1991; Davis, 2009).

The species *B. oleracea* is cultivated with many crop types, including broccoli, cauliflower, cabbages, Chinese kale and kohlrabi. Young plants of these crops may lose the growing shoot, a phenomenon known as “blind” or “blindness” which prevents the production of a marketable product. The occurrence of blind *B. oleracea* plants has been described already in the 1940s. It is characterized by termination of leaf primordia initiation in the SAM (Forsyth et al., 1999). It has been reported that depending on the moment of exposure to the inductive conditions and the developmental stage of the plant, five to ten leaves can be formed before the SAM ceases (Wiebosch et al., 1950). The last formed leaf may have a normal shape or consists of a petiole only, lacking a leaf blade (Forsyth et al., 1999). In a previous study (Wiebosch et al., 1950), three forms of blindness have been distinguished: empty hearted plants (with a dent in the stem), needle types (forming a pin-like structure at the position of the SAM) and pitcher plants (pitcher-shaped leaf as last structure). For plant growers blind plants are problematic, because recognizing affected plants at an early stage before transplanting them into the field is hardly possible, resulting in high economic losses that can be up to 95% in broccoli (Wurr et al., 1996). During 70 years of research, aiming to identify potential causes, various growth and environmental conditions have been proposed to induce blindness, including low temperature during early stages of development (Salter, 1957), freezing conditions (Mounsey-Wood, 1957), low solar radiation (Wurr et al., 1996), sowing date (Wurr et al., 1996), and molybdenum deficiency (Agarwala, 1952). However, overall the results are inconclusive and no main and common cause of blindness induction has been described. Besides environmental effects, there is likely a genetic component involved because growers experience that some varieties are more susceptible to blindness than others under seemingly the same environmental conditions.

To study and elucidate the basis of SAM arrest in *B. oleracea*, it is essential to have a reproducible induction system for blindness under controlled conditions. The induction method used by Wurr et al. (1996), with low light intensities is time and space consuming as blind plants develop several leaves prior to the loss of SAM activity. Furthermore, this method has shown to work for broccoli only (*B. oleracea* convar. *botrytis*). Therefore, we aimed to establish an early blindness induction system, useful for a spectrum of *B. oleracea* crop types and to apply this method to study the morphological, physiological and genetic mechanisms underlying SAM arrest in *B. oleracea* plants.

## Materials and Methods

### Plant Material

To develop a protocol for blindness induction, we used various *B. oleracea* seed lots from our laboratory collection of seed samples. These seed lots, left from previous seed research projects, represented several crop types. Seed lot numbers (in our collection), crop type and sensitivity for blindness are given in Supplementary Table S1. The AGDH doubled haploid population (Bohuon et al., 1996), was used to study genetic variation in sensitivity for blindness induction. This population was developed by crossing two double haploid parents, a rapid-cycling Chinese kale line, *B. oleracea* var. *alboglabra* (A12DHd), and a Calabrese broccoli line, *B. oleracea* convar. *botrytis* var. *italica* (GDDH33), through microspore culture of the F_1_. Seeds from about 100 double haploid lines were multiplied by seed companies in The Netherlands at three different locations under protected cultivation with natural light conditions.

Subsequent experiments, to study morphological effects and gene expression, were performed with seeds from the sensitive seed lot 1645 from the green cabbage cultivar Stanton F1 and specific lines from the AGDH population.

### Induction of Blindness in *B. oleracea*

To develop an assay for detecting sensitivity to the induction of blindness, the seeds were imbibed at low temperatures for a period between one and 14 days in darkness. Dry seeds were sown in Petri dishes on a layer of three filter papers moistened with cold demineralized water and incubated at the tested induction temperature in the dark. Temperature was measured with data loggers (EL-USB-2, Lascar Electronics, United Kingdom) next to the Petri dishes. In the first experiments temperatures between 0 and 10.5°C were tested. As the lowest temperatures gave the strongest induction, a temperature between 0 and 1°C was used in most of the subsequent experiments. This was achieved by placing the Petri dishes with seeds in a Styrofoam box filled with melting ice. The box with seeds and ice was placed in an incubator at 4°C. Cold induction for the QTL analysis with a large number of lines required mores space and a cold room at 1.5°C was used. After the cold incubation the seeds were transferred to individual pots of a 240 pot tray (pot size 2.8 cm × 2.8 cm × 4 cm, Modiform, Leusden, The Netherlands) filled with washed coco-peat and placed in a growth chamber at 20°C with 18 h incandescent light (150 μmol). Moisture was provided to the seedlings with tap water during the first 2 weeks and a standard nutrient solution was applied in the 3rd week. Plants were scored by eye for the absence of a growing shoot 3 weeks after sowing, unless stated otherwise. Seedling behavior under non-inducing (control) conditions was studied by imbibing dry seeds directly at 20°C.

### Microscopy

For histological analysis the material was fixed in 4% paraformaldehyde for a maximum of 16 h. Afterward, the samples were transferred to 30% ethanol overnight, than dehydrated in steps of 30 min until incubation in 96% of ethanol. Subsequently, the Technovit protocol was followed according to the manufacturer (Heraeus Kulzer GmbH, Wehrheim, Germany). The Technovit blocks were cut with a microtome in sections of 3 μm and toluidine blue was used for staining. Digital pictures were made with the Zeiss Axioskop microscope. Images were taken with a Leica DFC320 digital camera attached to the microscope and processed using Adobe Photoshop for white balance and contrast correction. Electron microscopy pictures were made at the Electron Microscopy Centre using the FEI Magellan FESEM high resolution scanning electron microscope.

For imaging nuclei with DNA replication activity, seeds were taken from the cold induction assay and directly placed on sterile growth medium (Daishin agar, Duchefa Biochemie) with Murashige and Skoog medium, Gamborg B5 Vitamins (Duchefa Biochemie) containing 10 μM EdU (Invitrogen) and germinated in a growth cabinet under 24 h light and at 17°C. After 48 h treatment time, shoot apices were dissected, dehydrated, and stained as described in Schiessl et al. (2012). The sections were imaged with a confocal laser scanning microscope (Leica SPE DM5500 upright microscope) using Leica Application Suite Advanced Fluorescence software (version 1.8.2, Leica Microsystems CMS, Mannheim, Germany).

### QTL Mapping

For QTL mapping, double haploid lines of the AGDH population (Bohuon et al., 1996; Sebastian et al., 2000) were studied. For this analysis, seeds from the whole population were reproduced at three locations with up to five plants per line per location. Seeds from the individual plants were kept separate. Lines that turned out to be phenotypically impure were discarded from the analysis. As a result, from the original 117 AGDH lines a subset of 85 lines were analyzed. Due to the large numbers of seeds to be tested, a slightly adjusted induction protocol was used. During the cold induction seeds were sown in the grooves of folded filter paper placed in plastic (15 cm × 21 cm) trays and moistened with ice-cold water. The trays with seeds were incubated in a cold room at 1.5°C for 10 days. After the cold induction the seeds were transferred to pots in the greenhouse as described above. Seedling evaluation was 3 weeks later. The experiment was performed in a randomized block setup with two blocks, and seeds from all individual plants represented in every block, with 12 seeds per mother plant as experimental unit. GenStat (2014) and Payne et al. (2014) was used for a multi-environment QTL analysis. For this analysis the genetic map as published by Broadley et al. (2008) was used. This map is based on the work of (Sebastian et al., 2000). The natural logarithm of the proportion of the blind plants per combination of line and location was used for analysis. Only combinations with at least 25 seedlings were used in the analysis. For testing the significance of a QTL, the method of (Li and Ji, 2005) was employed to calculate the significance threshold assuming a genome-wide significance level of 5%.

### Identification of QTL Region

The physical contig corresponding to the C03 QTL region was identified by first determining the genomic region (on chromosomes C03) that could be amplified with the SSR primers flanking the QTL region using *in silico* PCR^1^. Then finally, the QTL was chosen as the smallest genomic region encapsulated by SSR amplicons which included all markers with LOD score ≥ 2.5. The QTL on chromosome C03 was determined to start at position 18434598 and end at 34773929 as defined by the amplified regions corresponding to the SSR markers: *BoSF2985* and *boa011*. A total of 1354 genes are predicted to be encoded in this QTL-region.

### Transcriptome Analysis by RNA-seq

Total RNA was isolated at different time points, in triplicate after the induction treatment, from meristem enriched plant material (ten plants for each sample; lines AG 1020 and AG 5010) using the Spin Plant RNA Mini Kit from Invitrap^®^ (Stratec Molecular, Berlin, Germany). RNA samples were treated with DNAse I. Sequencing libraries were prepared with the TruSeq RNA Sample Preparation kit (Illumina, San Diego, CA, USA) following the manufacturer manual. Paired-end sequencing was done on an Illiumina Hi-Seq 2000 machine with 100 bp read length.

RNA-seq reads were mapped against the genome of *B. oleracea*^2^ using TopHat version 2.0.12 (Kim et al., 2013) with the following settings: -N 5 –read-edit-dist 5 –read-gap-length 3. HTSeq-count (Anders et al., 2014) was used for counting the number of reads mapping to annotated gene. Differential gene expression analyses were performed using DESeq2 (Love et al., 2014).

We assigned Gene ontology (GO) terms to *B. oleracea* genes by performing similarity searches against the predicted proteome of *A. thaliana* version TAIR10^3^ using BlastP (Altschul et al., 1990). The GO terms were inherited from the best blast hit (*e*-value < = 1e^-5^). The GO term annotation was formatted such that it could be used for enrichment analysis using BINGO (Maere et al., 2005). The background gene set for each enrichment analysis consisted of all genes expressed (read count > 0) in at least one of the samples being compared.

### RNA-Seq Data Accession Numbers

The raw RNA-seq sequence reads were deposited at the NCBI Sequence Read Archive (SRA) under the BioProject number: PRJNA319618.

### Gene Expression Analysis by qRT-PCR

Homologs of *A. thaliana* genes active in the SAM were identified in *B. oleracea* by Blast (blastp) searches. Alignments of the best hits from the blast search were made using ClustalW (Thompson et al., 1994). Subsequently, primers were designed for the *B. oleracea* genes with highest overall sequence homology to the *Arabidopsis* genes. For a selection of genes identified to be differentially expressed based on the RNA-seq analyses, primers were designed using the corresponding predicted coding sequences. Primer sequences and homology percentages can be found in Supplementary Table S2. Finally the uniqueness of the generated primers was tested using *in silico* PCR. RNA for the qRT-PCR experiments was isolated from meristem enriched tissue of ten plants per biological replicate that were dissected under a microscope at the desired time-point in triplicate. Total RNA was isolated from this material as described above for the RNA-seq. Afterward, cDNA was synthesized from 1 μg of total RNA using the TaqMan reverse transcriptase kit (Roche). Gene expression was measured by quantitative RT-PCR (qRT-PCR) with four biological replicates, using SYBR green mix from Bio-Rad, and making use of the primer combinations indicated in Supplementary Table S2. The single copy *BoYLS8* gene was used as reference gene for normalization and data were analyzed using the ΔΔCT method (Livak and Schmittgen, 2001; Schmittgen and Livak, 2008).

## Results

### Imbibition at Low Non-freezing Temperature Can Induce Blindness in Susceptible Seed Lots

**FIGURE 1:**
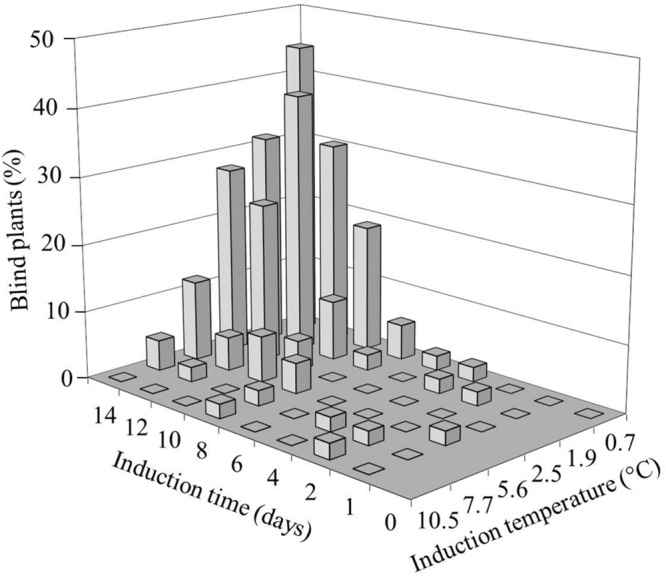
**Effect of temperature and induction time on the frequency of *Brassica oleracea* plants with arrested shoot apical meristem (SAM; blind plants).** Seeds of the cultivar Stanton (white cabbage) were imbibed for different durations at various induction temperatures, followed by transfer to 20°C. The seedling phenotype was scored 3 weeks after transfer.

We investigated the role and effect of low (non-freezing) temperatures on *B. oleracea* SAM loss during seedling development. To induce blindness, seeds were imbibed in darkness at temperatures ranging from 0.7 to 10.5°C for one to 14 days (**Figure 1**). After 10 days of exposing seeds to cold incubation below 11°C, none had reached the phase of radicle protrusion, which occurred for most seeds within 24 h after transfer of the seeds from the cold to 20°C. After 14 days of cold incubation at 10.5°C some seeds showed radicle protrusion. The blind seedlings obtained from these cold treated seed batches exhibited a gradual variation in the severity of blindness, with either no leaves at all or a few aberrant leaves (Supplementary Figure S1). A clear relation between severity of blindness and length of the treatment was observed: the lower temperature and longer treatment durations induced higher frequencies of blind seedlings. Incubation around 0–1°C proved to be the best induction temperature for an assay to analyze sensitivity, since it resulted in the most severe induction without affecting germination capacity. Extending this induction period at the lowest temperatures to 15 or 20 days strongly decreased the number of emerging seedlings.

To test whether there is a general mechanism involved and to understand if the assay developed here can be used to screen a wide range of *B. oleracea* varieties, we tested four different genotypes (**Table 1**). The assay conditions resulted in general in a reduction of 10–20% in seed germination, but with a clear induction of blind plants in all four seed lots tested, showing that our induction method is applicable for several *B. oleracea* crop types (green cabbage, kohlrabi, and broccoli). Subsequently, this result was confirmed with a larger number of seeds lots (Supplementary Table S1).

**Table 1:** Response of three seed lots from *Brassica oleracea* cultivars and one double haploid line from the AGDH population, to standard growth conditions (control) or after induction by treating the seeds with low temperature for 10 days on moist filter paper at 0–1°C.

Seed lot number	*B. oleracea* type	Seedlings obtained		Frequency blind plants	
		Control	Induced	Control	Induced
1645	Green cabbage (Stanton F1)	94%	75%	0%	63%
2105	Kohlrabi	92%	85%	0%	80%
2110	Broccoli	96%	79%	8%	55%
2518	AG 1012(DH line)	89%	79%	0%	29%

*Per seed lot 53 seeds were used. After the pre-incubation, the seeds were sown in coco peat. Not all seeds resulted in seedlings, mostly because of germination failure. The frequency of blind plants was analyzed 3 weeks after transfer.*

### Leaf Number and Architecture Is Aberrant in Blind *B. oleracea* Seedlings

**FIGURE 2:**
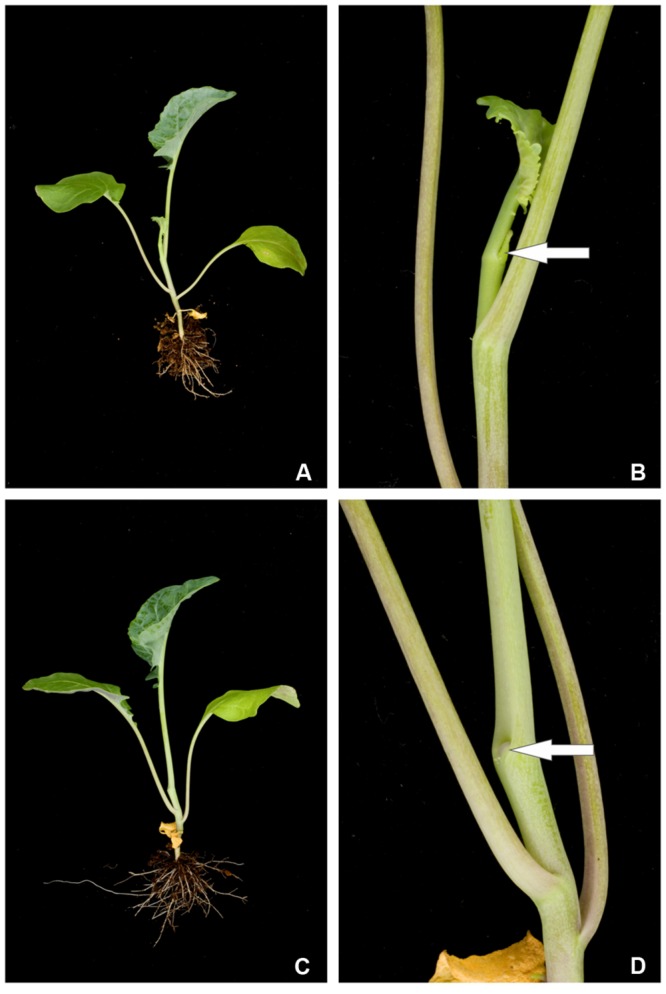
**Phenotype of a 4 weeks old normal (non-blind) *B. oleracea* plant **(A)** and **(B)** compared to a blind plant of the same seed lot with an arrested SAM **(C)** and **(D)**.** The arrows shown in the enlargements **(B)** and **(D)** point to the position of the SAM or where it used to be.

In general stem cells in the *B. oleracea* SAM give rise to leaf primordia, one every few days. These primordia grow out to show a normal seedling with leaves along the stem in different stages of development. With blind seedlings this normal situation is disrupted and less leaves are formed, or aberrations in leaf architecture are observed. We analyzed the phenotype of 4 weeks old seedlings macroscopically and an example of a non-blind (normal) and blind plant is depicted in **Figure 2**. The non-blind plant showed three expanded leaves, besides two small developing leaves emerging from the apex (**Figures 2A,B**). In contrast, the blind plant had developed three expanded leaves only and stopped the production of subsequent leaves (**Figures 2C,D**). Often the morphology of the non-blind and blind plants was very similar at this particular stage, but further leaf development was arrested in blind plants. At the position where new leaves usually emerge the arrested plant showed a dent and exhibited absence of a growing tip and further leaf initiation (**Figure 2D**). We observed that the stage of development at which the arrest of the meristem occurs is variable since blind plants differed in their appearance from no leaves to plants with a few leaves, although these leaves can often be aberrantly shaped (Supplementary Figure S1). We also noticed that some arrested seedlings had the ability to generate new meristems from the axils of their cotyledons (Supplementary Figure S1C) and these axillary meristems could develop into proper shoots that continued growth and development.

While comparing the germination behavior of blind seedlings to non-blind ones, we observed that some of the blind seedlings showed reduced root growth or lost their root meristematic activity (Supplementary Figure S2). As there were also blind seedlings with a normal growing root, it seems that reduced activity of the root apical meristem is not fully linked to a lack of shoot development (Supplementary Figure S2C).

### Blind Plants Have Disorganized Shoot Apical Meristems

**FIGURE 3:**
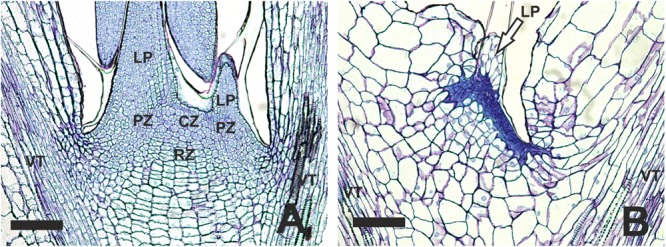
**Histological differences observed in the SAM area of *B. oleracea* (Line AG5010) seedlings at 6 days after the induction treatment. (A)** Seedling with a non-blind phenotype. **(B)** Seedling with a blind phenotype. CZ, Central zone; LP, Leaf primordium; PZ, Peripheral zone; RZ, Rib zone; VT, Vascular tissue reaching into the cotyledons. In **(B)** the arrow points to what may be the remnants of a leaf primordium initiated during seed development. The scale bars represent 100 μm.

To examine more closely the SAM area of blind plants, we performed histological analysis of normal and blind seedlings without visible leaves. Sections of 6-day old non-blind seedlings showed all three zones of a typical SAM: the central zone, the periphery zone and the rib zone (**Figure 3A**). The central zone displayed a group of small cells comprising the stem cells. The rib-zone showed the typical parallel file structure of cells (Jürgens, 1995). In the center of the cotyledons and in the main stem vasculature was observed. Vasculature was also visible in the sections of the blind seedlings, indicating that the sections were made approximately at the same position in the seedlings. In comparison to the non-blind seedling, the blind seedlings lacked a clear structure of the central and rib zone (**Figure 3B**). In general, the cell pattern of the blind seedlings was less organized and the small meristematic cells were replaced by less and large parenchyma-like cells. No clear organized L1 layer was visible in the blind seedlings at the position of the SAM in non-blind seedlings.

**FIGURE 4:**
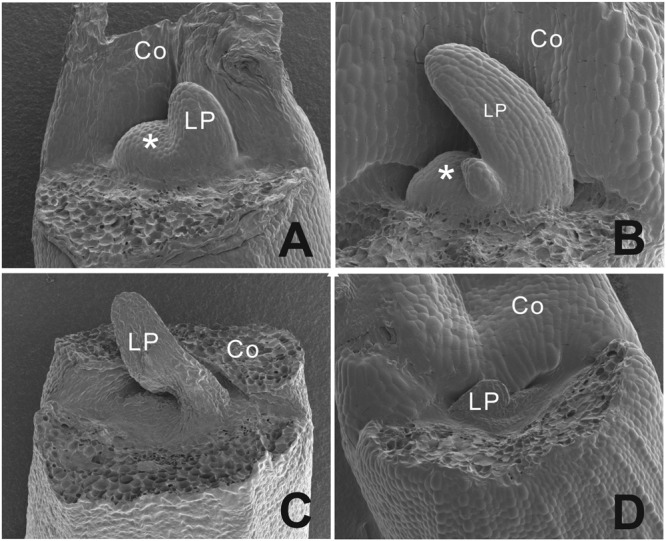
**Scanning electron microscopy picture of the phenotypic difference between a functional normal and arrested SAM in *B. oleracea* seedlings from cultivar Stanton F1. (A)** Three days old seedlings with a functional normal SAM and one leaf developing. **(B)** Four days old seedling with one leaf emerging and functional normal SAM. **(C)** Three days old seedling with an aberrant SAM area and one leaf-like structure. **(D)** Four days old seedling with an aberrant SAM area. Asterisks indicate the SAM, Co, cotyledons; LP, leaf primordium.

To analyze the phenotype at an earlier stage, scanning electron microscopy pictures were taken (**Figure 4**). We observed a dome shaped meristem in non-blind seedlings with emerging leaf primordia at the flanks of the dome (**Figures 4A,B**), while blind seedlings showed a flattened area at the position of the SAM with one leaf primordium in this case (**Figures 4C,D**).

### A QTL for Seedling Blindness Is Identified on Linkage Group C3

**FIGURE 5:**
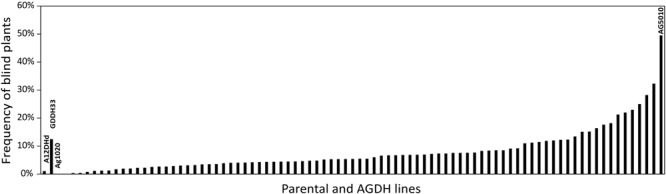
**Genetic variance in sensitivity to blindness after cold induction.** Percentage of blind seedlings per line from the AGDH population after exposed to blindness inducing conditions for 10 days. The parental lines of the population A12DHd and GDDH33 are indicated, as well as the lines AG1020and AG5010 that were used in further analyses. Results represent the mean percentage of blind plants from three repetitive analyses with on average 87 seeds per line per analysis. Due to limited availability of parental line seeds these were only analyzed twice, with on average 54 (for A12DHd) and 33 (GDDH33) seeds. Approximately 80% of the seeds resulted in seedlings that could be evaluated.

**FIGURE 6:**
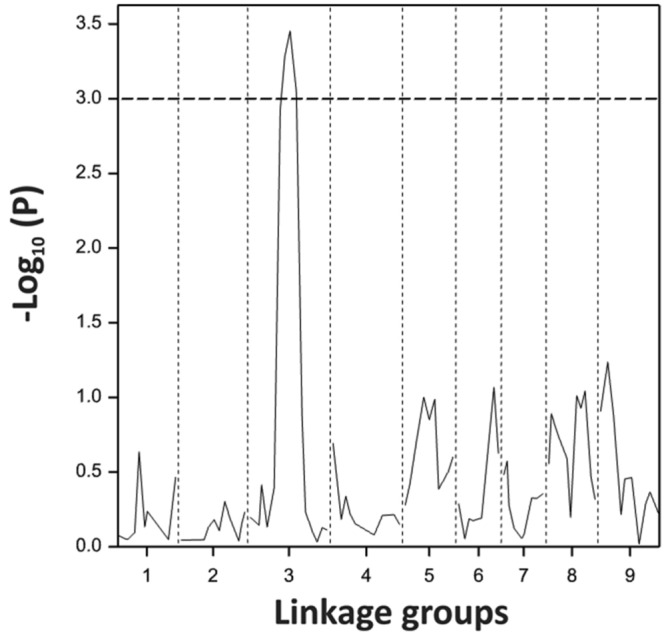
**Probability score for sensitivity to blindness induction for the *B. oleracea* linkage group C3 analyzed with 92 lines of the AGDH population.** Dotted line represents the threshold given by the permutation testing.

Using the blindness assay described above, we determined the sensitivity for blindness in the AGDH *B. oleracea* mapping population. A large variation was observed between lines in sensitivity to blindness induction (**Figure 5**). The sensitivity of some of the offspring DH lines in the AGDH population appeared to be even higher than that of the parents, indicating transgressive segregation for blindness under inducing conditions. In the ANOVA analysis no block effect was observed (*P* = 0.65), but an effect of genetic variation (*P* < 0.001, 25% of the variance explained) and an effect of seed production location (*P* < 0.001, 11% variance explained) were observed (**Table 2**). The latter indicates a significant effect of seed production on the sensitivity of the seeds. One QTL was identified on linkage group C03 (**Figure 6**). The QTL analysis further showed that the allele responsible for increased sensitivity to blindness induction is from the rapid-cycling Chinese kale parental line (A12DHd).

**Table 2:** QTL effects for the three seed production locations for the sensitivity to induction of blind plants as observed in the AGDH lines obtained from the cross GDDH33 × A12DHHd.

Seed production location	QTL effect	High value allele from	Standard error	*P*-value	Percentage variance explained
Company 1	0.41	A12DHHd	*0.172*	0.018	12.2
Company 2	0.17	A12DHHd	*0.139*	0.217	2.0
Company 3	0.58	A12DHHd	*0.142*	0.000	19.0

### Transcriptional Differences in Blind and Non-blind *B. oleracea* Plants

**FIGURE 7:**
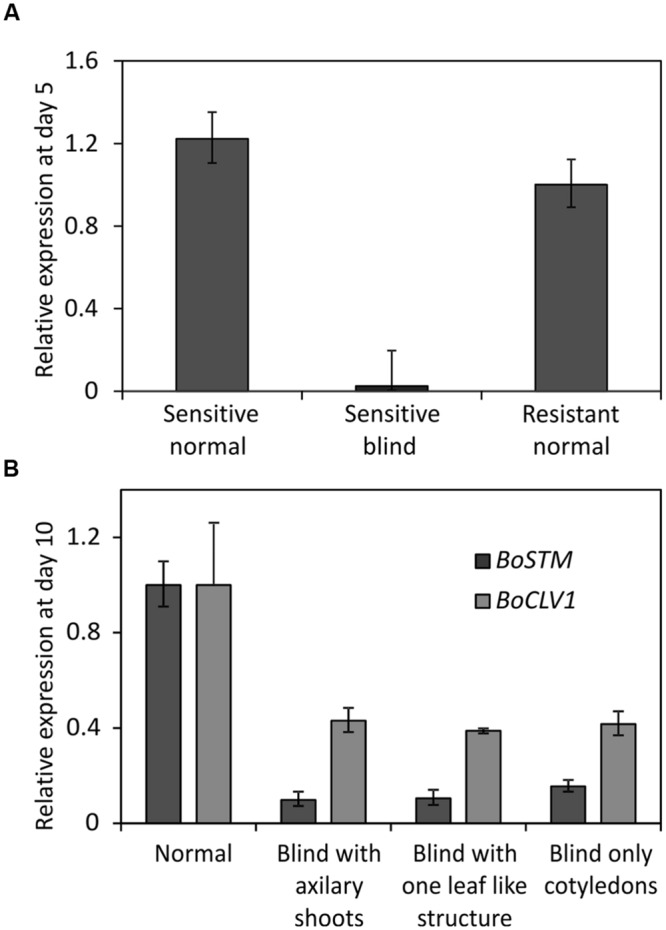
**(A)** Relative expression of *BoSTM* (orthologous to *STM* in *Arabidopsis thaliana*) at day 5 after finishing the cold treatment to induce blindness. Expression levels measured by Q-PCR using meristem enriched tissue from non-blind looking seedlings (Sensitive non-blind) and blind looking seedlings (Sensitive blind) from the sensitive genotype (line AG5010) and from non-blind seedlings (Resistant non-blind) from a resistant genotype (line AG1020). Error bars represent the standard error of four biological replicates. **(B)** Expression of *BoSTM* in seedlings of different phenotype at day 10 after the cold induction of seeds from line AG5010 (a sensitive genotype). ‘Normal’ represents the expression of plants with a meristem. ‘Blind with axillary shoots’ represents plants that lost their SAM but develop side shoots in the axils of the cotyledons. ‘Blind with leaf like structure’ represent the expression of plants that produced one leaf-like structure before arresting the meristem. ‘Blind only cotyledons,’ represents plants that only developed cotyledons and no side-shoots. Expression is provided in comparison to the resistant non-blind seedlings. Error bars represent the standard error of four biological replicates.

Two lines of the AGDH population were selected for analysis of gene expression. Line AG1020, showing a low amount of blindness after cold treatment and therefore, was considered as tolerant. Line AG5010, showing a high frequency of blind seedlings after cold induction and was therefore considered as sensitive (**Figure 5**). Seedlings from those lines were used to identify genome-wide differentially expressed genes associated to blindness. To get an indication at what time-point after the cold induction the SAM loses its meristematic cells, we first performed expression measurements of the gene *SHOOT MERISTEMLESS (STM)*, because it encodes a class-I KNOTTED-like homeobox transcription factor that is expressed throughout the SAM (Long et al., 1996; Rupp et al., 1999; Scofield et al., 2007) and is supposed to have a role in preventing stem cell differentiation (Lenhard et al., 2002; Williams and Fletcher, 2005). Quantitative RT-PCR, showed that at day 5 after the cold induction the expression of *BoSTM* is significantly decreased in blind seedlings compared to non-blind seedlings from the sensitive line AG5010, as well as compared to the seedlings from the resistant line AG1020 (**Figure 7A**). Additionally, we measured the expression of *BoSTM* in 10 days old seedlings after the cold induction (**Figure 7B**). Based on these results we selected day 2 after the cold induction as first time point for RNA-seq analysis. Unfortunately, plants are too small at this time point to determine their phenotype and hence, RNA samples are a mix of ‘to become’ non-blind and ‘to become’ blind seedlings (approximately 50%). In order to have also pure samples of blind and non-blind seedlings, we collected also material at day 7 after the cold induction, which is the earliest moment to distinguish flawlessly between blind and non-blind plants. RNA-seq was done on this material and differential gene expression was determined (adjusted *p*-value ≤ 0.01 and at least a twofold change). To eliminate genes that are solely cold-responsive at day 2, we eliminated those genes that were significantly up- or down-regulated in both the sensitive and resistant genotypes upon the cold treatment. For time point 7 days after induction the comparison was made between sensitive induced blind seedlings and non-blind seedlings. These filter criteria resulted in the identification of 2962 and 5929 statistically significant differentially expressed genes at day 2 (Supplementary Table S3) and day 7 (Supplementary Table S4), respectively. The quality of the RNA-seq data set was assessed by qRT-PCR for a selected number of identified differentially expressed genes (Supplementary Figure S3). Subsequently, we performed an analysis to identify GO terms over-represented (*p*-value < 0.05 after Benjamini Hochberg correction) in these selected gene sets at 2 and 7 days after the cold induction. This analysis revealed that at day 2, especially genes related to photosynthesis and cell proliferation were differentially expressed (Supplementary Table S5), whereas at day 7 genes involved in nuclear and chromosomal organization and cell proliferation were overrepresented among the differentially expressed genes in blind seedlings (Supplementary Table S6).

To get a further indication of genome-wide differentially expressed genes that might be related to meristem development and hence, the occurrence of blindness, we made use of a published *Arabidopsis* gene expression dataset for the various SAM regions (Yadav et al., 2009). In this study, genes were identified that are expressed in cells specifically marked by *CLAVATA3 (CLV3)* and *WUSCHEL (WUS)* expression, which represent meristematic cells, and cells marked by *FILAMENTOUS FLOWER (FIL)*, representing differentiating cells at the flanks of the SAM. Potential functional homologs of these genes were identified in our RNA-seq dataset and their behavior was analyzed. As can be seen in Supplementary Table S7 cold affected a higher number of genes potentially expressed in the central zone of the SAM (meristematic class genes) of a blindness-sensitive line than of a resistant line, 2 days after the treatment. As expected, this concerns more down-regulation then up-regulation in comparison to the resistant plants. A similar trend can be seen at day 7, a correlation between what is happening at the morphological and the molecular level (gene expression) during the development of blindness.

In total, 90 and 160 of the genes within the C03 QTL were differentially expressed at days 2 and 7, respectively. Most homologs of the genes present in the top 20 most significantly differentially expressed genes in the CO3 QTL region (**Tables 3** and **4**) are expressed in the *Arabidopsis* vegetative meristem and/or in the seed (during imbibition) according to previously published micro-array data (Winter et al., 2007). Only for one gene, gBol042544, no *Arabidopsis* homolog could be identified. Homologs of genes coding for the Minichromosome maintenance family proteins 4 and 5 (*MCM4, MCM5)*, Minichromosome maintenance family protein *PROLIFERA (PRL), A. THALIANA MutS HOMOLOG* 2 and 6 *(MSH2, MSH6)* and a HOMOLOG OF NUCLEOLAR PROTEIN 56 (*NOP56*) are differentially expressed when comparing samples from sensitive blind and sensitive non-blind seedlings at both time points.

**Table 3:** Top 20 genes located in the QTL region with the most significant difference between the induced sensitive sample relative to the un-induced sensitive sample 2 days after the cold induction.

*B. oleracea* annotation	Homolog in *Arabidopsis thaliana*	TAIR annotation	Log_2_ fold change	Adjusted *P*-value
gBol042544	-	-	1.867	2.61E-07
gBol042553	AT2G07690	MCM5 Minichromosome maintenance family protein	-1.329	1.41E-03
gBol035542	AT3G13650	Disease resistance-responsive family protein	1.294	1.99E-03
gBol006646	AT5G61810	Mitochondrial substrate carrier family protein	1.879	7.37E-03
gBol022897	AT3G18280	Bifunctional inhibitor/lipid-transfer protein/seed storage 2S albumin superfamily protein	1.94	1.09E+02
gBol035492	AT3G12860	NOP56-like pre RNA processing ribonucleoprotein	-1.713	1.27E+02
gBol035512	AT3G13080	ATMRP3, MRP3, ABCC3 multidrug resistance-associated protein 3	-1.51	1.74E+02
gBol030686	AT4G02060	PRL Minichromosome maintenance family protein	-1.274	1.51E+03
gBol035455	AT3G12280	RBR1 retinoblastoma-related 1	-1.382	1.52E+03
gBol015863	AT5G64420	DNA polymerase V family	-1.343	1.55E+04
gBol030685	AT4G02070	MSH6, MSH6-1, ATMSH6 MutS homolog 6	-1.225	1.92E+04
gBol010776	AT4G01150	Unknown protein	1.392	2.73E+04
gBol026646	AT3G20630	UBP14, TTN6, ATUBP14, PER1 ubiquitin-specific protease 14	-1.05	8.65E+04
gBol026545	AT3G22660	rRNA processing protein-related	-1.42	1.37E
gBol026635	AT3G21055	PSBTN photosystem II subunit T	1.72	2.25E
gBol022890	AT3G18524	MSH2, ATMSH2 MutS homolog 2	-1.166	2.89E
gBol026595	AT3G21720	ICL isocitrate lyase	-1.295	3.31E
gBol012448	AT2G17720	2-oxoglutarate (2OG) and Fe(II)-dependent oxygenase superfamily protein	1.311	3.42E
gBol042665	AT2G16440	MCM4 Minichromosome maintenance family protein	-1.284	4.23E

*The annotation of the genes in *B. oleracea, A. thaliana*, and their TAIR annotations are indicated.*

**Table 4:** Top 20 genes located in the QTL region with the most significant difference between the induced sensitive sample relative to the un-induced sensitive sample seven after the cold induction.

*B. oleracea* annotation	Homolog in *Arabidopsis thaliana*	Go annotation	Log_2_ fold change	Adjusted *P*-value
gBol035557	AT3G13960	Growth-regulating factor 5	-5.758	1.10E^-52^
gBol042608	AT2G14900	Gibberellin-regulated family protein	1.849	5.88E^-36^
gBol030685	AT4G02070	MSH6, MSH6-1, ATMSH6 MutS homolog 6	-1.92	9.47E^-22^
gBol042665	AT2G16440	MCM4 Minichromosome maintenance family protein	-1.884	2.02E^-18^
gBol030686	AT4G02060	PRL Minichromosome maintenance family protein	-1.956	4.26E^-17^
gBol010669	AT3G02480	Late embryogenesis abundant protein (LEA) family protein	8.359	4.95E^-17^
gBol022964	AT3G17010	AP2/B3-like transcriptional factor	-2.869	2.44E^-16^
gBol042553	AT2G07690	MCM5 Minichromosome maintenance family protein	-1.755	6.57E^-16^
gBol035492	AT3G12860	NOP56-like pre RNA processing ribonucleoprotein	-2.183	1.91E^-15^
gBol042690	AT2G16890	UDP-Glycosyltransferase superfamily protein	3.608	3.35E^-15^
gBol022890	AT3G18524	MSH2, ATMSH2 MutS homolog 2	-1.69	6.85E^-15^
gBol022986	AT3G16360	AHP4 HPT phosphotransmitter 4	2.756	8.65E^-11^
gBol042483	AT3G25190	Vacuolar iron transporter (VIT) family protein	1.745	9.47E^-10^
gBol010741	AT3G02000	ROXY1 Thioredoxin superfamily protein	-3.762	2.42E^-9^
gBol035580	AT3G14415	Aldolase-type TIM barrel family protein	-1.328	8.16E^-9^
gBol035541	AT3G13640	ATRLI1, RLI1 RNAse l inhibitor protein 1	-2.48	1.02E^-8^
gBol010957	AT3G10690	GYRA DNA GYRASE A	-1.409	2.26E^-8^
gBol035497	AT3G12970	Unknown protein	-2.438	2.70E^-8^
gBol035513	AT3G13130	Unknown protein;	6.022	1.14E^-7^

*The annotation of the genes in *B. oleracea, A. thaliana*, and their GO annotations are indicated.*

### In Blind *B. oleracea* Plants the Cell Cycle and DNA Replication is Disturbed

**FIGURE 8:**
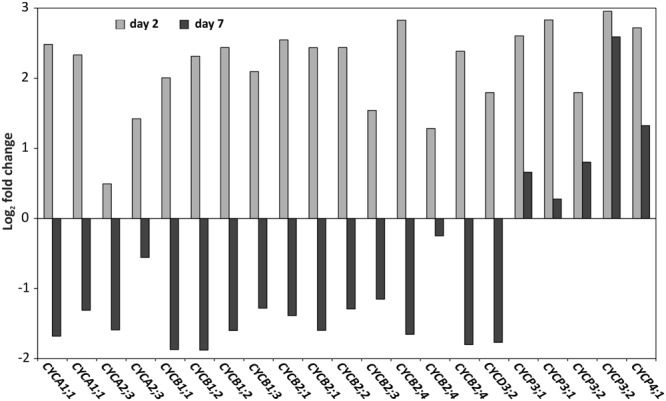
**Cyclins are inversely differentially expressed at days 2 and 7.** Log_2_ expression fold change of cyclin genes: *CYCA1;*1 (gBol021328, gBol043233/AT1G44110.1),*CYCA2;3* (gBol038155, gBol009873/AT1G15570.1),*CYCB1;1* (gBol028907/AT4G37490.1),*CYCB1;2* (gBol024500,gBol015440/AT5G06150.1),*CYCB1;3* (gBol023203/AT3G11520.1),*CYCB2;1* (gBol012439, gBol007505/AT2G17620.1),*CYCB2;2* (gBol018699/AT4G35620.1) CYCB2;3 (gBol006997/AT1G20610.1), *CYCB2;3* (gBol006997/AT1G20610.1), *CYCB2;4* (gBol039865, gBol015175, gBol027646/AT1G76310.1), *CYCD3;2* (gBol027246/AT5G67260.1), *CYCP3;1* (gBol029526,gBol030150/AT2G45080.1), *CYCP3;2* (gBol045616,gBol001087/AT3G60550.1) and *CYCP4;1* (gBol029520/AT2G44740.1). Day 2 (gray) is the comparison between the samples of the induced sensitive (AG5010) compared to the induced resistant seedlings (AG1020). Day 7 (black) is the comparison between samples of the induced sensitive (AG5010) blind and induced sensitive non-blindnormal (AG5010) seedlings.

Since we identified GO-terms related to cell proliferation as being over-represented both at days 2 and 7 after cold treatment in seedlings from a blindness-sensitive line (AG5010; Supplementary Tables S5 and S6), we further focused on cell cycle related genes in the genome-wide transcriptome data set. For cell division or mitosis, DNA needs to be duplicated in the S-Phase of the cell cycle. Cyclins represent key genes in this process (Inzé and De Veylder, 2006) and surprisingly, a number of them were up-regulated 2 days after the cold induction in the sensitive genotype compared to the resistant one (**Figure 8**). However, the same set of genes showed down-regulation in the sensitive line 7 days after cold induction, except for three P-Type cyclins, which remained their higher expression levels also at day 7 (**Figure 8**).

**FIGURE 9:**
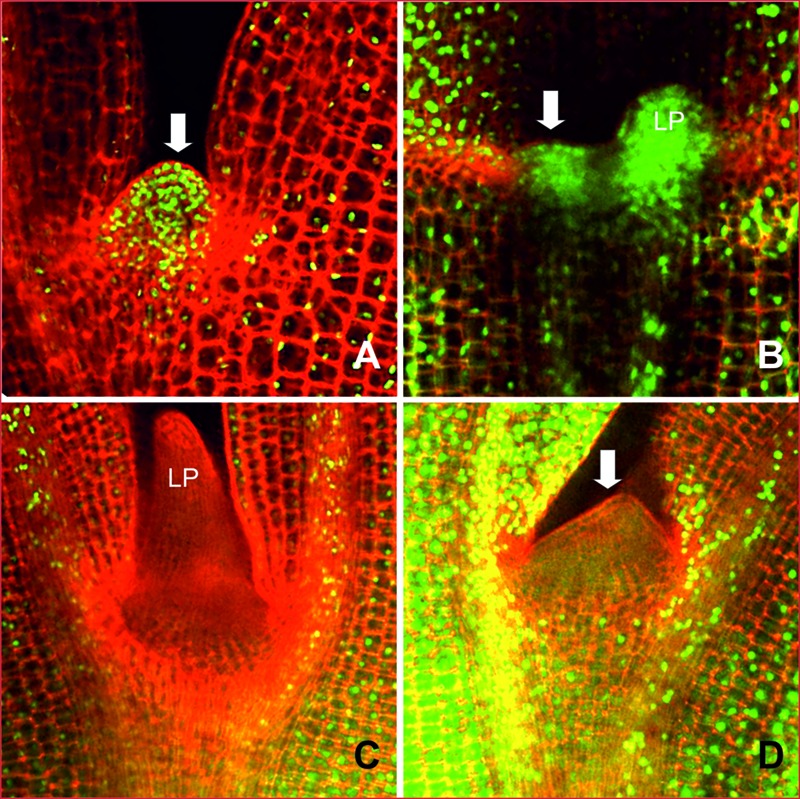
**DNA replication activity in the meristematic region of *B. oleracea* seedlings from line AG5010 and AG1020 2 days after the cold treatment.** The plant material was stained with EdU to visualize nuclei with DNA replication activity, indicated by green fluorescence. **(A)** and **(B)** Represent seedlings from the blindness induction treatment resistant line (AG1020). **(C)** and **(D)** Representative seedlings from the blindness induction sensitive line (AG5010) that develops SAM arrest after the cold induction. LP, Leaf primordium; LS, Leaf-like structure, arrows point at the apical dome, missing in **(C)**.

To analyze the cell division activity *in situ* we placed seeds from the resistant line AG1020 and the sensitive line AG5010 after the cold induction, on growth medium containing EdU, a chemical that is incorporated in DNA during replication. Two days after the cold induction EdU incorporation was visible as green fluorescence in nuclei from the resistant line (**Figures 9A,B**) and in all visible tissues: the SAM, hypocotyl and cotyledons. In the seedling from the sensitive line, DNA replication was observed in the hypocotyl and cotyledon tissues, but the nuclei in the SAM area did not incorporate EdU, demonstrating that blindness is related to an absence of DNA replication (**Figures 9C,D**). DNA replication was also abolished in the leaf primordium of the sensitive line compared to the resistant one (compare **Figures 9C,B**).

In contrast to the set of cyclin genes (**Figure 8**), which are not located in the C03 QTL region and are most likely indirectly affected by the blindness phenomenon, a number of other cell cycle related genes are located in the QTL region. *RETINOBLASTOMA RELATED (RBR)* gBol035455, a plant homolog of the tumor suppressor gene identified in animals (Friend et al., 1986), is lower expressed at both analyzed time points in the sensitive background in comparison to the resistant one and is located in the C03 QTL region (Supplementary Table S3 and S4).

Other genes present in the C03 QTL region (**Tables 3** and **4**) and related to the cell cycle are members of the *Mini Chromosome Maintenance (MCM) 2-3-5* gene family. Genes of this family play important roles in both the initiation and the elongation phase of eukaryotic DNA replication (Tye, 1994). Furthermore, *BoMSH2* and *BoMSH6* are also located in the CO3 QTL region and significantly differentially expressed. Interestingly, an interaction between the *MSH* genes and cell cycle control has been suggested (Lario et al., 2011).

## Discussion

### Cold Imbibition Leads to Stem Cell Differentiation

Under field conditions the frequency of blind plants varies and can be induced at different stages of plant development. Blindness has been reported to occur after plants have developed several leaves (Wiebosch et al., 1950; Forsyth et al., 1999). We focused in this study on the early occurrence of blindness during germination and seedling establishment. We found that low temperature stress is a major trigger for SAM arrest in sensitive *B. oleracea* seedlings, since a high frequency of blind plants could be induced by about 10 days of incubation of fully imbibed seeds at 0–1°C. Also Forsyth et al. (1999) found in their study that a period of cold and low light intensities followed by a warmer period with higher light intensities can lead to blindness in broccoli. In contrast to their method, our induction system is faster and induces blindness in young seedlings compared to 8 weeks old plants in the broccoli protocol. Additionally, our induction system proved to be applicable for seed lots from several *B. oleracea* crop types, while the previously published protocol was tested with one broccoli cultivar only.

As is also observed in practice, not all seeds from a particular genotype or seed lot exposed to the inducing conditions developed blindness. Furthermore, the degree of meristematic arrest varies between seedlings. The reason for this variation in blindness within a seed lot could lie in differences between seeds for the perception or processing of the signal in the meristem. Alternatively, the cold treatment might gradually damage the meristematic cells in the SAM in an accumulating manner. An indication for this latter hypothesis is that a longer cold incubation reduces the germination capacity of the seeds and increases the frequency of blindness.

The histological analysis showed that the SAM region of blind seedlings comprised more differentiated cells than meristematic cells when compared to a non-blind seedling and the normal tunica-corpus structure of the SAM (Steeves and Sussex, 1989; Meyerowitz, 1997) is missing in the arrested seedlings. A similar observation has been made in sections from much older blind broccoli shoots that also lack small sized meristematic cells (Forsyth et al., 1999).

The development of blind plants is associated with lack or reduction in DNA replication in the SAM area and loss of its meristematic activity. Genetic variation for sensitivity to SAM arrest was demonstrated and a QTL was identified in a segregating population. We also showed that seed production conditions influenced the sensitivity of the seed lots. Based on a genome-wide expression analysis, we found a number of potential candidate genes in the QTL region with significantly altered expression levels.

### A Genetic Factor Affects Blindness Sensitivity

The QTL analysis in a *B. oleracea* DH population from a cross between broccoli and Chinese kale showed that blindness has a genetic component and that the genetic variation for blindness sensitivity can be partly explained by alleles located on linkage group C03. This finding opens possibilities to breed for blindness insensitive genotypes. Subsequently, we started to unravel the molecular mechanism underlying the development of blindness by combining the information of the QTL region on C03 and genome-wide differential expression analysis. Our molecular analyses revealed that in blind *B. oleracea* seedlings – which resemble the phenotype of *stm* knock-out mutants in *Arabidopsis* (Long et al., 1996) – expression of the *B. oleracea STM* homolog *BoSTM* was down regulated. This observation suggests that the cells of the SAM in the arrested seedlings are differentiating and gradually losing their meristematic capacity. Additionally, *BoSTM* was also lower expressed in blind seedlings that developed only one leaf or in blind seedlings forming side-shoots, showing that the molecular network of stem cell maintenance is affected in the various blind phenotypes. The axillary shoots observed in some blind plants developed further and remained indeterminate, taking over the function of the SAM. These axillary ‘escape’ shoots are also observed in *Arabidopsis wus* mutants (Laux et al., 1996) and this phenotype suggests that the molecular mechanism underlying the cold treatment is specific for the cells in the SAM, while the axillary buds are released from growth repression after loss of the SAM. This is in line with the fact that axillary bud outgrowth is inhibited by the presence of the main shoot (‘apical dominance’) and that in the absence of the SAM, lateral meristem outgrowth is triggered (Cline, 1994).

In our genome-wide transcriptome analysis cell cycle genes were up-regulated 2 days after the cold induction in sensitive genotypes compared to the resistant genotypes. However, at day 7 after the cold induction the same genes showed strongly reduced expression levels in the sensitive genotype compared to the resistant one. This strong reduction in expression at the later time point indicates that the cell cycle is arrested in the sensitive seedlings and no new cells are formed in the SAM region. The arrest of the cell cycle was also confirmed by EdU staining that marks nuclei in the S-Phase, when the dye is incorporated in DNA during DNA replication (Schiessl et al., 2012). Specifically, the nuclei in the SAM showed lower staining in the sensitive genotypes compared to the resistant ones 2 days after cold treatment. Based on this result we can conclude that the up-regulation of cyclin genes at day 2 in the sensitive genotype does not lead to increased DNA synthesis and cell division activity, on the contrary, the meristematic cells do not enter the S-Phase in the blind seedlings. During normal cell-cycle progression different types of cyclins show maximum expression at specific time-points of the phases of the cell cycle (G0/G1-S-G2-M; Bloom and Cross, 2007). While *CYCD3* peaks at the G2/M phase, e.g., *CYCA3* reaches its highest expression levels at the S-phase of the cell-cycle. Therefore, the massive up-regulation at day 2 of different types of cyclins in the sensitive genotype could be related to the blind phenotype. It is possible that the tightly controlled expression of the cell cycle genes in normal dividing cells is lost and as a result many *CYC* genes are miss-expressed in the sensitive genotype. Whether the expression changes of the above discussed cell-cycle-related genes are a secondary effect or the primary cause of the blindness phenotype is difficult to determine. It is more likely that that they are indirectly affected in the blind seedlings, because *CYC* genes are absent in the list of differentially expressed genes located in the QTL region.

Another major regulator of the cell cycle is *RBR*, which shows lower expression levels in the sensitive genotype at days 2 and 7 after the cold induction and more interestingly, the gene is located in the QTL region. It has been shown that decreased *RBR* expression causes arrest of plant development and acts especially on stem cell maintenance in the SAM (Borghi et al., 2010). A lack of *RBR* expression alters the meristem activity by disruption of the CLAVATA-WUSCHEL feedback loop. In a similar way the miss-expression of *RBR* in the sensitive genotypes, observed here, could lead to the arrest of the *CLAVATA-WUSCHEL* feedback loop, resulting in cell differentiation and cell-cycle arrest reminiscent with the phenotype of the *wus* mutants.

Desiccation tolerant seeds enter a quiescent stage at the end of seed-maturation. After water uptake the embryo has to re-establish its active state and commences cell division and growth. These processes are well organized in time, for instance, DNA repair precedes DNA replication (van Pijlen et al., 1996) to repair eventual damage caused by desiccation or rehydration. DNA damage keeps the cyclin-dependent kinase (CDKA)-cyclin (CYCD) complex in a phosphorylated and therefore inactive state; in that state it fails to phosphorylate RBR and the cell is prohibited in entering the S-Phase of the cell cycle (Nasmyth, 1995; Novak et al., 1998). Un-phosphorylated RBR is bound by E2F transcription factors, which in its turn cannot activate the *MSH* genes involved in DNA mismatch repair (Nakagami et al., 1999, 2002; Uemukai et al., 2005; Yadav et al., 2009). Several *MSH* genes show differential expression at both time points and are located in the QTL region. Other interesting candidate genes that are differentially expressed and present in the QTL region are members of the *MCM* gene family. The proteins encoded by these genes, first discovered in yeast, but present in all eukaryotic genomes, form a helicase complex that has a role in both the initiation and the elongation phases of eukaryotic DNA replication (Tye, 1994). Consistent with a role in DNA replication, *PROLIFERA (PRL)* a member of the *MCM* genes from *Arabidopsis* (Springer et al., 2000) is preferentially expressed in young tissues that contain a high number of replicating cells, like embryos, young organs and meristems. A knock-out mutant of the *PRL* gene is embryo-lethal demonstrating its essential role in cell division and tissue growth. A gene encoding for PRL Minichromosome maintenance family protein is located on the QTL and differentially expressed in blind and non-blind seedlings. It is conceivable to hypothesize that the down-regulation of the *MCM* and *RBR* homologs in sensitive *B. oleracea* plants underlies the arrest of cell division and hence loss of meristematic activity in the SAM.

## Conclusion

Our study revealed that blindness in *B. oleracea* seedlings is related to arrest of cells in the SAM or part of them. An important trigger for the induction of blindness in sensitive seed lots turned out to be low temperatures during germination. We observed that seed production conditions can influence the sensitivity of seed lots. The sensitivity to the occurrence of blind seedlings has also a genetic basis and we discovered a QTL on linkage group C03. Through a genome-wide transcriptome analysis we narrowed the amount of possible candidate genes to 90 and found that especially genes involved in the cell cycle were miss-regulated. Therefore, we think that two genes belonging to the *MCM* gene family, a *RBR* gene, a key and several *MutS homologs* (*MSH*), all located in QTL region, are the most likely candidates for being involved in the development of blindness in sensitive genotypes.

## Author Contributions

JJ, JK, RI, and SG contributed to the design of the research; performance of the research; data analysis, collection, or interpretation; and writing the manuscript. ES contributed to the bioinformatics analysis. GB and GA contributed to the design of the experiments, data interpretation and writing the manuscript. All authors have read and approved the manuscript.

## Conflict of Interest Statement

The authors declare that the research was conducted in the absence of any commercial or financial relationships that could be construed as a potential conflict of interest.

The authors declare that the research was 50% financed by the Dutch government and a cash (25%) and in-kind (25%) contribution by a consortium of companies (Bejo Seeds, ENZA Seeds, Incotec, Nickerson-Zwaan, Nunhems, Monsanto Holland, Syngenta Seeds, Rijk Zwaan and the association of Dutch plant raisers organized in Plantum NL).

## Footnotes

1 http://hgwdev.cse.ucsc.edu/~kent/exe/linux/

2 http://www.ocri-genomics.org/bolbase/

3 http://www.arabidopsis.org
