# Supplementary material for: Low Temperature Affects Stem Cell Maintenance in Brassica oleracea Seedlings
Source: Front Plant Sci. 2016 Jun 8;7:800. doi: 10.3389/fpls.2016.00800 (PMC4896912; doi:10.3389/fpls.2016.00800)
Supplement: Supplementary file 1 [file Table_1.PDF]

Supplemental material to:
de Jonge J, Kodde J, Severing EI, Bonnema G, Angenent GC, Immink RGH and Groot SPC (2016) Low Temperature Affects Stem Cell Maintenance in Brassica oleracea Seedlings. Front. Plant Sci. 7:800. doi: 10.3389/fpls.2016.00800

Supplemental Table S1. Response of various *B. oleracea* seed lots, retrieved from our seed physiology seed sample collection, to low temperature pre-incubation for 8 days on moist filter paper at 2°C. Per seed lot 51 seeds were used. After an eight days cold (2 °C) and moist pre-incubation, the seeds were sown in coco peat. Not all seeds resulted in seedlings, mostly because of germination failure. The frequency of blind plants was analysed 20 days after sowing. For several seed lots the crop type or variety is unknown.

Seed lot number in WUR Bioscience Seed Research collection	*B. oleracea* crop type	seedlings obtained	frequency blind plants
2516	kohlrabi	94%	81%
2105	kohlrabi	92%	66%
2110	broccoli	90%	61%
1645	green cabbage (Stanton F1)	86%	36%
2518	unknown	86%	23%
13	kohlrabi	84%	16%
1405	unknown	88%	16%
2107	kohlrabi	94%	15%
2262	broccoli	94%	15%
53	kohlrabi	14%	14%
2108	kohlrabi	84%	14%
12	unknown	59%	7%
2114	broccoli	92%	4%
1727	unknown	94%	4%
2517	unknown	78%	3%
39	kohlrabi	80%	2%
2263	broccoli	94%	2%
2111	cauliflower	98%	2%
1	unknown	45%	0%
38	kohlrabi	88%	0%
46	white cabbage	71%	0%
1401	unknown	96%	0%
1726	unknown	96%	0%
2109	broccoli	98%	0%
2112	cauliflower	94%	0%
