# Supplementary material for: Low Temperature Affects Stem Cell Maintenance in Brassica oleracea Seedlings
Source: Front Plant Sci. 2016 Jun 8;7:800. doi: 10.3389/fpls.2016.00800 (PMC4896912; doi:10.3389/fpls.2016.00800)
Supplement: Supplementary file 2 [file Table_2.PDF]

Supplemental material to:
de Jonge J, Kodde J, Severing EI, Bonnema G, Angenent GC, Immink RGH and Groot SPC (2016) Low Temperature Affects Stem Cell Maintenance in Brassica oleracea Seedlings. Front. Plant Sci. 7:800. doi: 10.3389/fpls.2016.00800

**Supplemental Table S2.** Primer sequences used for rtPCR. Gene name abbreviations from A. thaliana and ATG numbers from A. thaliana. Brassica ID: Homologous genes in Brassica and their homology percentage on nucleotide level, if the exact gene sequence were not known.

| TAIR AtG-code | Gene symbol | Bol code | Identity at nt level | Forward | Reverse |
|---|---|---|---|---|---|
| AT1G62360 | STM | Bol011007 | 89% | TGATGGTCCGATGTGTCCTA | GCACCAGAGGAAGGAGAACA |
| AT1G75820 | CLV1 | Bol027692 | 82 | CCGGCTCGATCCCAATCGGAATCGG | AAGGAAGTGTCGTTGAAGACTAGG |
| AT3G18730 | BRU1 | Bol002997 | 87% | AAGGATCTGCTGCCTACGTG | CGGTGTCGTTGTTCTCTTCA |
| AT3G22380 | TIC | Bol026557 | 83% | CAACTGGTTCACCAATGCAG | GTGGTTTTGGAGACCGAAGA |
| AT2G38530 | LTP2 | Bol017820 | 81% | TAAACAACATGGCCCGTACA | TGGTGGTTTTGCTGATCTTG |
| AT2G02100 | - | Bol007171 | 91% | CCACAGGTATGGGTCCAGTC | GGAAACCACGGCATTTACC |
| AT3G20150 | - | Bol026667 | 86% | ACGCGCAGATAAAGCTGAAG | AAACCTGGCATGACCAAGAA |
