# Supplementary material for: Low Temperature Affects Stem Cell Maintenance in Brassica oleracea Seedlings
Source: Front Plant Sci. 2016 Jun 8;7:800. doi: 10.3389/fpls.2016.00800 (PMC4896912; doi:10.3389/fpls.2016.00800)
Supplement: Supplementary file 3 [file Table_3.PDF]

Supplemental material to:
de Jonge J, Kodde J, Severing EI, Bonnema G, Angenent GC, Immink RGH and Groot SPC (2016) Low Temperature Affects Stem Cell Maintenance in Brassica oleracea Seedlings. Front. Plant Sci. 7:800. doi: 10.3389/fpls.2016.00800

Supplemental Table S3. RNA-seq analysis of gene expression after 2 days in response to cold treatment for genes in the identified QTL region . Listed here are genes located in the identified QTL region and whose expression is changed only in the sensitive genotype and not in the resistant genotype to the response of the cold treatment. Values are presented in fragments per kilobase of exon per million fragments mapped (FPMK). Only reads with significant difference (P-value 0.05) are presented. BRAD represents the gene code from the Brassica Database (http://brassicadb.org/brad/), ATG represents the gene code for the *Arabidopsis thaliana* genes (http://www.arabidopsis.org/). Data for all genes differentially expressed at day 2 are presented in a separate supplemental Excel file (see supplemental table S3A).

Bol-ID	Log2 Fold-Change	Adjusted P-value	Best Arabidopsis hit	Description
gBol042544	1.9	2.61E-21		no hit
gBol042553	-1.3	1.41E-17	AT2G07690.1	Symbols: MCM5 | Minichromosome maintenance (MCM2/3/5) family protein | chr2:3523379-3527388 REVERSE LENGTH=727
gBol035542	1.3	1.99E-17	AT3G13650.1	Symbols: | Disease resistance-responsive (dirigent-like protein) family protein | chr3:4463056-4463616 FORWARD LENGTH=186
gBol006646	1.9	7.37E-17	AT5G61810.1	Symbols: | Mitochondrial substrate carrier family protein | chr5:24831843-24833735 REVERSE LENGTH=478
gBol042665	-1.3	4.23E-15	AT2G16440.1	Symbols: MCM4 | Minichromosome maintenance (MCM2/3/5) family protein | chr2:7126536-7130665 REVERSE LENGTH=847
gBol026545	-1.4	1.37E-14	AT3G22660.1	Symbols: | rRNA processing protein-related | chr3:8016237-8017118 REVERSE LENGTH=293
gBol022890	-1.2	2.89E-14	AT3G18524.1	Symbols: MSH2, ATMSH2 | MUTS homolog 2 | chr3:6368151-6372409 REVERSE LENGTH=937
gBol026595	-1.3	3.31E-14	AT3G21720.1	Symbols: ICL | isocitrate lyase | chr3:7652789-7655873 REVERSE LENGTH=576
gBol026635	1.7	2.25E-13	AT3G21055.1	Symbols: PSBTN | photosystem II subunit T | chr3:7376761-7377072 REVERSE LENGTH=103
gBol022897	1.9	1.09E-12	AT3G18280.1	Symbols: | Bifunctional inhibitor/lipid-transfer protein/seed storage 2S albumin superfamily protein | chr3:6267102-6267392 FORWARD LENGTH=96
gBol035492	-1.7	1.27E-12	AT3G12860.1	Symbols: | NOP56-like pre RNA processing ribonucleoprotein | chr3:4091678-4093921 FORWARD LENGTH=499
gBol035512	-1.5	1.74E-12	AT3G13080.1	Symbols: ATMRP3, MRP3, ABCC3 | multidrug resistance-associated protein 3 | chr3:4196019-4201250 REVERSE LENGTH=1514
gBol012448	1.3	3.42E-12	AT2G17720.1	Symbols: | 2-oxoglutarate (2OG) and Fe(II)-dependent oxygenase superfamily protein | chr2:7697513-7699174 FORWARD LENGTH=291
gBol030686	-1.3	1.51E-11	AT4G02060.2	Symbols: PRL | Minichromosome maintenance (MCM2/3/5) family protein | chr4:901484-905297 FORWARD LENGTH=716
gBol035455	-1.4	1.52E-10	AT3G12280.2	Symbols: RBR1 | retinoblastoma-related 1 | chr3:3913671-3918433 REVERSE LENGTH=1012
gBol015863	-1.3	1.55E-10	AT5G64420.1	Symbols: | DNA polymerase V family | chr5:25756416-25761122 FORWARD LENGTH=1306

gBol030685	-1.2	1.92E-10	AT4G02070.1	Symbols: MSH6, MSH6-1, ATMSH6 | MUTS homolog 6 | chr4:906079-912930 FORWARD LENGTH=1324
gBol010776	1.4	2.73E-10	AT4G01150.1	Symbols: | unknown protein; FUNCTIONS IN: molecular_function unknown; INVOLVED IN: biological_process unknown; LOCATED IN: thylakoid, chloroplast thylakoid membrane, chloroplast, plastoglobule, chloroplast envelope; EXPRESSED IN: 23 plant structures; EXPRESSED DURING: 14 growth stages; BEST Arabidopsis thaliana protein match is: unknown protein (TAIR:AT4G38100.1); Has 323 Blast hits to 323 proteins in 59 species: Archae - 0; Bacteria - 107; Metazoa - 0; Fungi - 0; Plants - 206; Viruses - 0; Other Eukaryotes - 10 (source: NCBI BLink). | chr4:493692-494668 FORWARD LENGTH=164
gBol026646	-1.1	8.65E-10	AT3G20630.1	Symbols: UBP14, TTN6, ATUBP14, PER1 | ubiquitin-specific protease 14 | chr3:7203001-7208340 REVERSE LENGTH=797
gBol022972	-1.7	2.11E-09	AT3G16770.1	Symbols: RAP2.3, ATEBP, ERF72, EBP | ethylene-responsive element binding protein | chr3:5705784-5706768 FORWARD LENGTH=248
gBol035508	-1.1	3.33E-09	AT3G13070.1	Symbols: | CBS domain-containing protein / transporter associated domain-containing protein | chr3:4191511-4195112 REVERSE LENGTH=661
gBol026580	1.2	7.69E-09	AT3G22142.1	Symbols: | Bifunctional inhibitor/lipid-transfer protein/seed storage 2S albumin superfamily protein | chr3:7803604-7808046 REVERSE LENGTH=1480
gBol030668	-1.1	8.26E-09	AT4G02390.1	Symbols: APP, PARP1, ATPARP1, PP | poly(ADP-ribose) polymerase | chr4:1050104-1053960 FORWARD LENGTH=637
gBol035576	3.1	1.38E-08	AT3G14310.1	Symbols: ATPME3, PME3 | pectin methylesterase 3 | chr3:4772214-4775095 REVERSE LENGTH=592
gBol006660	2.3	3.08E-08	AT3G49530.1	Symbols: ANAC062, NTL6, NAC062 | NAC domain containing protein 62 | chr3:18362639-18364717 REVERSE LENGTH=469
gBol042507	1.0	3.80E-08	AT1G54410.1	Symbols: | dehydrin family protein | chr1:20310305-20310601 REVERSE LENGTH=98
gBol015853	1.4	3.92E-08	AT5G64310.1	Symbols: AGP1, ATAGP1 | arabinogalactan protein 1 | chr5:25722018-25722413 FORWARD LENGTH=131
gBol042568	1.3	5.70E-08	AT2G12400.1	Symbols: | unknown protein; FUNCTIONS IN: molecular_function unknown; INVOLVED IN: biological_process unknown; LOCATED IN: endomembrane system; EXPRESSED IN: 25 plant structures; EXPRESSED DURING: 13 growth stages; BEST Arabidopsis thaliana protein match is: unknown protein (TAIR:AT2G25270.1); Has 177 Blast hits to 172 proteins in 23 species: Archae - 0; Bacteria - 2; Metazoa - 3; Fungi - 0; Plants - 164; Viruses - 0; Other Eukaryotes - 8 (source: NCBI BLink). | chr2:5005144-5008140 REVERSE LENGTH=541
gBol035490	1.1	7.13E-08	AT3G12800.1	Symbols: SDRB, DECR | short-chain dehydrogenase-reductase B | chr3:4063463-4064757 REVERSE LENGTH=298
gBol030661	2.7	1.11E-07	AT4G02520.1	Symbols: ATGSTF2, ATPM24.1, ATPM24, GST2, GSTF2 | glutathione S-transferase PHI 2 | chr4:1110673-1111531 REVERSE LENGTH=212
gBol026647	2.2	1.51E-07	AT3G20600.1	Symbols: NDR1 | Late embryogenesis abundant (LEA) hydroxyproline-rich glycoprotein family | chr3:7194877-7195536 FORWARD LENGTH=219
gBol035500	-1.4	1.53E-07	AT3G12980.1	Symbols: HAC5, ATHPCAT4 | histone acetyltransferase of the CBP family 5 | chr3:4146919-4154495 FORWARD LENGTH=1670
gBol026654	1.3	1.87E-07	AT3G20510.1	Symbols: | Transmembrane proteins 14C | chr3:7160884-7161991 FORWARD LENGTH=119

gBol035454	-1.1	4.68E-07	AT3G12270.1	Symbols: ATPRMT3, PRMT3 | protein arginine methyltransferase 3 | chr3:3910642-3913122 FORWARD LENGTH=601
gBol041293	1.5	5.38E-07	AT3G44260.1	Symbols: | Polynucleotidyl transferase, ribonuclease H-like superfamily protein | chr3:15952213-15953055 REVERSE LENGTH=280
gBol022915	2.2	8.43E-07	AT1G48440.1	Symbols: | B-cell receptor-associated 31-like | chr1:17907075-17908327 FORWARD LENGTH=129
gBol035489	1.4	8.89E-07	AT3G12780.1	Symbols: PGK1 | phosphoglycerate kinase 1 | chr3:4061127-4063140 REVERSE LENGTH=481
gBol022940	1.2	1.02E-06	AT3G17365.1	Symbols: | S-adenosyl-L-methionine-dependent methyltransferases superfamily protein | chr3:5947144-5948766 REVERSE LENGTH=239
gBol042396	-1.3	1.02E-06	AT3G16030.1	Symbols: CES101 | lectin protein kinase family protein | chr3:5439609-5442802 FORWARD LENGTH=850
gBol035522	1.2	1.03E-06	AT3G13310.1	Symbols: | Chaperone DnaJ-domain superfamily protein | chr3:4310827-4311300 REVERSE LENGTH=157
gBol006649	-1.0	1.16E-06	AT5G61780.1	Symbols: Tudor2, AtTudor2, TSN2 | TUDOR-SN protein 2 | chr5:24822012-24826641 FORWARD LENGTH=985
gBol030677	-1.6	1.24E-06	AT4G02170.1	Symbols: | unknown protein; BEST Arabidopsis thaliana protein match is: unknown protein (TAIR:AT5G38700.1); Has 53 Blast hits to 53 proteins in 10 species: Archae - 0; Bacteria - 0; Metazoa - 0; Fungi - 0; Plants - 53; Viruses - 0; Other Eukaryotes - 0 (source: NCBI BLink). | chr4:958126-958641 FORWARD LENGTH=171
gBol012478	-1.9	1.46E-06	AT2G18220.1	Symbols: | Noc2p family | chr2:7928254-7931851 FORWARD LENGTH=764
gBol023011	3.5	2.21E-06	AT3G15870.1	Symbols: | Fatty acid desaturase family protein | chr3:5362911-5364425 FORWARD LENGTH=361
gBol015899	-2.2	4.46E-06	AT5G64750.1	Symbols: ABR1 | Integrase-type DNA-binding superfamily protein | chr5:25891679-25893656 FORWARD LENGTH=391
gBol006676	1.3	5.79E-06	AT3G49220.1	Symbols: | Plant invertase/pectin methylesterase inhibitor superfamily | chr3:18249840-18253647 FORWARD LENGTH=598
gBol010714	1.4	6.89E-06	AT3G01510.1	Symbols: LSF1 | like SEX4 1 | chr3:198855-201682 REVERSE LENGTH=591
gBol010742	2.6	6.98E-06	AT4G00050.1	Symbols: UNE10 | basic helix-loop-helix (bHLH) DNA-binding superfamily protein | chr4:17863-19848 FORWARD LENGTH=399
gBol035565	2.9	8.58E-06	AT3G14190.1	Symbols: | unknown protein; BEST Arabidopsis thaliana protein match is: unknown protein (TAIR:AT5G12360.1); Has 18 Blast hits to 18 proteins in 5 species: Archae - 0; Bacteria - 0; Metazoa - 0; Fungi - 0; Plants - 18; Viruses - 0; Other Eukaryotes - 0 (source: NCBI BLink). | chr3:4710907-4711790 FORWARD LENGTH=193
gBol042468	1.4	1.13E-05	AT3G24730.1	Symbols: | mRNA splicing factor, thioredoxin-like U5 snRNP | chr3:9030152-9030894 REVERSE LENGTH=159
gBol010760	1.7	1.47E-05	AT4G00700.1	Symbols: | C2 calcium/lipid-binding plant phosphoribosyltransferase family protein | chr4:286260-289369 FORWARD LENGTH=1006
gBol030660	1.6	1.63E-05	AT2G02930.1	Symbols: ATGSTF3, GST16, GSTF3 | glutathione S-transferase F3 | chr2:851348-852106 REVERSE LENGTH=212
gBol023009	-2.0	1.66E-05	AT3G15880.3	Symbols: WSIP2 | WUS-interacting protein 2 | chr3:5364792-5371869 REVERSE LENGTH=1125
gBol042560	-1.1	1.99E-05	AT2G06210.1	Symbols: ELF8, VIP6 | binding | chr2:2429108-2436588 REVERSE LENGTH=1091
gBol015871	-1.4	6.53E-05	AT5G64510.1	Symbols: TIN1 | unknown protein; FUNCTIONS IN: molecular_function unknown; INVOLVED IN: biological_process unknown; LOCATED IN: endomembrane system; EXPRESSED IN: 17 plant structures; EXPRESSED DURING: 10 growth

				stages; Has 35333 Blast hits to 34131 proteins in 2444 species: Archae - 798; Bacteria - 22429; Metazoa - 974; Fungi - 991; Plants - 531; Viruses - 0; Other Eukaryotes - 9610 (source: NCBI BLink). | chr5:25784592-25786152 FORWARD LENGTH=424
gBol041280	1.0	6.63E-05	AT3G44380.1	Symbols: | Late embryogenesis abundant (LEA) hydroxyproline-rich glycoprotein family | chr3:16036254-16036814 REVERSE LENGTH=186
gBol042472	-1.2	7.91E-05	AT3G24880.1	Symbols: | Helicase/SANT-associated, DNA binding protein | chr3:9086457-9095537 REVERSE LENGTH=1957
gBol026699	2.7	0.00011	AT3G19580.2	Symbols: AZF2, ZF2 | zinc-finger protein 2 | chr3:6803293-6804114 REVERSE LENGTH=273
gBol010786	1.8	0.00013	AT4G01010.1	Symbols: ATCNGC13, CNGC13 | cyclic nucleotide-gated channel 13 | chr4:434569-437242 REVERSE LENGTH=696
gBol022916	-1.8	0.00017	AT3G17710.1	Symbols: | F-box and associated interaction domains-containing protein | chr3:6055597-6056703 FORWARD LENGTH=368
gBol042431	2.3	0.00018	AT3G23920.1	Symbols: BAM1, BMY7, TR-BAMY | beta-amylase 1 | chr3:8641722-8644199 FORWARD LENGTH=575
gBol022954	1.1	0.00035	AT3G17120.2	Symbols: | unknown protein; BEST Arabidopsis thaliana protein match is: unknown protein (TAIR:AT1G02380.1); Has 35333 Blast hits to 34131 proteins in 2444 species: Archae - 798; Bacteria - 22429; Metazoa - 974; Fungi - 991; Plants - 531; Viruses - 0; Other Eukaryotes - 9610 (source: NCBI BLink). | chr3:5842410-5843246 FORWARD LENGTH=219
gBol012462	-1.0	0.00037	AT2G17930.1	Symbols: | Phosphatidylinositol 3- and 4-kinase family protein with FAT domain | chr2:7784455-7802230 REVERSE LENGTH=3858
gBol035458	-1.5	0.00038	AT3G12320.1	Symbols: | unknown protein; BEST Arabidopsis thaliana protein match is: unknown protein (TAIR:AT5G06980.4); Has 102 Blast hits to 102 proteins in 16 species: Archae - 0; Bacteria - 0; Metazoa - 0; Fungi - 0; Plants - 98; Viruses - 0; Other Eukaryotes - 4 (source: NCBI BLink). | chr3:3924034-3925262 FORWARD LENGTH=269
gBol022991	1.1	0.00040	AT3G16240.1	Symbols: DELTA-TIP, TIP2;1, DELTA-TIP1, AQP1, ATTIP2;1 | delta tonoplast integral protein | chr3:5505534-5506788 FORWARD LENGTH=250
gBol042440	1.4	0.00041	AT2G04400.1	Symbols: | Aldolase-type TIM barrel family protein | chr2:1531208-1533578 FORWARD LENGTH=402
gBol010767	1.5	0.00053	AT4G00770.1	Symbols: | unknown protein; Has 127 Blast hits to 120 proteins in 33 species: Archae - 0; Bacteria - 2; Metazoa - 6; Fungi - 8; Plants - 62; Viruses - 3; Other Eukaryotes - 46 (source: NCBI BLink). | chr4:331195-333050 FORWARD LENGTH=440
gBol012456	1.0	0.00154	AT2G17840.1	Symbols: ERD7 | Senescence/dehydration-associated protein-related | chr2:7755923-7757798 REVERSE LENGTH=452
gBol030644	2.8	0.00165	AT4G02800.1	Symbols: | unknown protein; FUNCTIONS IN: molecular_function unknown; INVOLVED IN: biological_process unknown; LOCATED IN: chloroplast; EXPRESSED IN: 16 plant structures; EXPRESSED DURING: 9 growth stages; BEST Arabidopsis thaliana protein match is: unknown protein (TAIR:AT5G01970.1); Has 3209 Blast hits to 2720 proteins in 308 species: Archae - 13; Bacteria - 213; Metazoa - 1207; Fungi - 247; Plants - 183; Viruses - 21; Other Eukaryotes - 1325 (source: NCBI BLink). | chr4:1250126-1251478 FORWARD LENGTH=333
gBol042464	-1.5	0.00167	AT3G24650.1	Symbols: ABI3, SIS10 | AP2/B3-like transcriptional factor family protein | chr3:8997911-9000780 FORWARD LENGTH=720

gBol012474	-1.1	0.00169	AT2G18193.1	Symbols: | P-loop containing nucleoside triphosphate hydrolases superfamily protein | chr2:7917621-7919184 REVERSE LENGTH=495
gBol026661	2.6	0.00181	AT3G20300.1	Symbols: | Protein of unknown function (DUF3537) | chr3:7079832-7081809 REVERSE LENGTH=452
gBol042670	2.3	0.00234	AT2G16580.1	Symbols: | SAUR-like auxin-responsive protein family | chr2:7186602-7186928 REVERSE LENGTH=108
gBol013800	1.6	0.00240	AT3G44960.1	Symbols: | unknown protein; Has 34 Blast hits to 34 proteins in 10 species: Archae - 0; Bacteria - 0; Metazoa - 3; Fungi - 0; Plants - 31; Viruses - 0; Other Eukaryotes - 0 (source: NCBI BLink). | chr3:16427795-16429151 FORWARD LENGTH=207
gBol022914	1.1	0.00306	AT3G17800.1	Symbols: | Protein of unknown function (DUF760) | chr3:6091248-6092873 REVERSE LENGTH=421
gBol010727	2.1	0.00332	AT3G01710.2	Symbols: | TPX2 (targeting protein for Xklp2) protein family | chr3:259952-261738 REVERSE LENGTH=388
gBol042587	1.7	0.00336	AT2G13790.1	Symbols: ATSERK4, SERK4, BKK1, BAK7 | somatic embryogenesis receptor-like kinase 4 | chr2:5741979-5746581 FORWARD LENGTH=620
gBol012439	2.5	0.00399	AT2G17620.1	Symbols: CYCB2;1 | Cyclin B2;1 | chr2:7664164-7666261 FORWARD LENGTH=429
gBol042405	-1.1	0.00405	AT3G23410.1	Symbols: ATFAO3, FAO3 | fatty alcohol oxidase 3 | chr3:8382860-8386024 FORWARD LENGTH=746
gBol030665	1.3	0.00422	AT4G08500.1	Symbols: MEKK1, ATMEKK1, MAPKKK8, ARAKIN | MAPK/ERK kinase kinase 1 | chr4:5404272-5407062 REVERSE LENGTH=608
gBol010938	1.3	0.00457	AT3G11230.2	Symbols: | Yippee family putative zinc-binding protein | chr3:3516683-3518193 FORWARD LENGTH=162
gBol041285	2.6	0.00484	AT3G44350.2	Symbols: anac061, NAC061 | NAC domain containing protein 61 | chr3:16022836-16024487 REVERSE LENGTH=241
gBol015932	1.3	0.00662	AT5G65140.1	Symbols: TPPJ | Haloacid dehalogenase-like hydrolase (HAD) superfamily protein | chr5:26019878-26022077 REVERSE LENGTH=370
gBol026683	-1.4	0.00708	AT3G19920.1	Symbols: | unknown protein; BEST Arabidopsis thaliana protein match is: unknown protein (TAIR:AT5G64230.1); Has 217 Blast hits to 217 proteins in 16 species: Archae - 0; Bacteria - 2; Metazoa - 0; Fungi - 0; Plants - 215; Viruses - 0; Other Eukaryotes - 0 (source: NCBI BLink). | chr3:6929840-6931771 REVERSE LENGTH=416
gBol035475	-1.0	0.00748	AT3G12590.1	Symbols: | unknown protein; INVOLVED IN: biological_process unknown; LOCATED IN: chloroplast; Has 50 Blast hits to 41 proteins in 15 species: Archae - 0; Bacteria - 0; Metazoa - 2; Fungi - 0; Plants - 43; Viruses - 0; Other Eukaryotes - 5 (source: NCBI BLink). | chr3:3996473-4003657 REVERSE LENGTH=1184
gBol022922	2.3	0.00766	AT3G17640.1	Symbols: | Leucine-rich repeat (LRR) family protein | chr3:6032393-6033583 FORWARD LENGTH=396
gBol042682	1.1	0.00783	AT4G35020.3	Symbols: RAC3 | RAC-like 3 | chr4:16673176-16674540 FORWARD LENGTH=198
gBol042557	1.4	0.00826	AT5G35320.1	Symbols: | unknown protein; Has 1807 Blast hits to 1807 proteins in 277 species: Archae - 0; Bacteria - 0; Metazoa - 736; Fungi - 347; Plants - 385; Viruses - 0; Other Eukaryotes - 339 (source: NCBI BLink). | chr5:13521893-13523144 FORWARD LENGTH=225
gBol012452	1.0	0.00895	AT2G17760.1	Symbols: | Eukaryotic aspartyl protease family protein | chr2:7713488-7716269 FORWARD LENGTH=513
gBol015922	1.4	0.00977	AT5G65030.1	Symbols: | unknown protein; BEST Arabidopsis thaliana protein match is: unknown protein (TAIR:AT5G10210.1); Has 1807 Blast hits to 1807 proteins in 277 species: Archae - 0; Bacteria - 0; Metazoa - 736; Fungi - 347; Plants - 385; Viruses - 0; Other Eukaryotes - 339 (source: NCBI BLink). | chr5:25975697-25976305 REVERSE LENGTH=202
