# Supplementary material for: Low Temperature Affects Stem Cell Maintenance in Brassica oleracea Seedlings
Source: Front Plant Sci. 2016 Jun 8;7:800. doi: 10.3389/fpls.2016.00800 (PMC4896912; doi:10.3389/fpls.2016.00800)
Supplement: Supplementary file 4 [file Table_4.PDF]

Supplemental material to:
de Jonge J, Kodde J, Severing EI, Bonnema G, Angenent GC, Immink RGH and Groot SPC (2016) Low Temperature Affects Stem Cell Maintenance in Brassica oleracea Seedlings. Front. Plant Sci. 7:800. doi: 10.3389/fpls.2016.00800

**Supplemental Table S4**. RNA-seq analysis of gene expression after 7 days in response to cold treatment. Listed are genes located in the identified QTL region and whose expression is changed only in the sensitive genotype and not in the resistant genotype to the response of the cold treatment. Values are presented in fragments per kilobase of exon per million fragments mapped (FPMK). Only reads with significant difference (P-value 0.05) are shown. BRAD represents the gene code from the Brassica Database (http://brassicadb.org/brad/), ATG represents the gene code for the *Arabidopsis thaliana* genes (http://www.arabidopsis.org/). Data for all genes differentially expressed at day 7 are presented in a separate supplemental Excel file (see supplemental table S4A),

| **Bol-ID** | **Log2 Fold-Change** | **Adjusted P-value** | **Best Arabidopsis hit** | **Description** |
|---|---|---|---|---|
| gBol035557 | -5.8 | 1.10E-65 | AT3G13960.1 | Symbols: AtGRF5, GRF5 \| growth-regulating factor 5 \| chr3:4608526-4610160 FORWARD LENGTH=397 |
| gBol042608 | 1.8 | 5.88E-50 | AT2G14900.1 | Symbols: \| Gibberellin-regulated family protein \| chr2:6404210-6405043 FORWARD LENGTH=108 |
| gBol030685 | -1.9 | 9.47E-36 | AT4G02070.1 | Symbols: MSH6, MSH6-1, ATMSH6 \| MUTS homolog 6 \| chr4:906079-912930 FORWARD LENGTH=1324 |
| gBol042665 | -1.9 | 2.02E-32 | AT2G16440.1 | Symbols: MCM4 \| Minichromosome maintenance (MCM2/3/5) family protein \| chr2:7126536-7130665 REVERSE LENGTH=847 |
| gBol030686 | -2.0 | 4.26E-31 | AT4G02060.2 | Symbols: PRL \| Minichromosome maintenance (MCM2/3/5) family protein \| chr4:901484-905297 FORWARD LENGTH=716 |
| gBol010669 | 8.4 | 4.95E-31 | AT3G02480.1 | Symbols: \| Late embryogenesis abundant protein (LEA) family protein \| chr3:512384-512857 FORWARD LENGTH=68 |
| gBol022964 | -2.9 | 2.44E-30 | AT3G17010.1 | Symbols: \| AP2/B3-like transcriptional factor family protein \| chr3:5800460-5802303 FORWARD LENGTH=302 |
| gBol042553 | -1.8 | 6.57E-30 | AT2G07690.1 | Symbols: MCM5 \| Minichromosome maintenance (MCM2/3/5) family protein \| chr2:3523379-3527388 REVERSE LENGTH=727 |
| gBol042690 | 3.6 | 3.35E-29 | AT2G16890.2 | Symbols: \| UDP-Glycosyltransferase superfamily protein \| chr2:7316938-7319022 FORWARD LENGTH=478 |
| gBol022890 | -1.7 | 6.85E-29 | AT3G18524.1 | Symbols: MSH2, ATMSH2 \| MUTS homolog 2 \| chr3:6368151-6372409 REVERSE LENGTH=937 |
| gBol035492 | -2.2 | 1.91E-28 | AT3G12860.1 | Symbols: \| NOP56-like pre RNA processing ribonucleoprotein \| chr3:4091678-4093921 FORWARD LENGTH=499 |
| gBol022986 | 2.8 | 8.65E-25 | AT3G16360.2 | Symbols: AHP4 \| HPT phosphotransmitter 4 \| chr3:5554351-5555518 FORWARD LENGTH=145 |
| gBol042483 | 1.7 | 9.47E-24 | AT3G25190.1 | Symbols: \| Vacuolar iron transporter (VIT) family protein \| chr3:9174505-9175614 FORWARD LENGTH=219 |
| gBol010741 | -3.8 | 2.42E-23 | AT3G02000.1 | Symbols: ROXY1 \| Thioredoxin superfamily protein \| chr3:332512-332922 REVERSE LENGTH=136 |
| gBol035580 | 1.3 | 8.16E-23 | AT3G14415.3 | Symbols: \| Aldolase-type TIM barrel family protein \| chr3:4818667-4820748 FORWARD LENGTH=367 |
| gBol035541 | -2.5 | 1.02E-22 | AT3G13640.1 | Symbols: ATRLI1, RLI1 \| RNAse l inhibitor protein 1 \| chr3:4458751-4461323 REVERSE LENGTH=603 |
| gBol010957 | -1.4 | 2.26E-22 | AT3G10690.1 | Symbols: GYRA \| DNA GYRASE A \| chr3:3339612-3346243 REVERSE LENGTH=950 |
| gBol035497 | -2.4 | 2.70E-22 | AT3G12970.1 | Symbols: \| unknown protein; BEST Arabidopsis thaliana protein match is: unknown protein (TAIR:AT1G56020.1); Has 2408 Blast hits to 418 proteins in 91 species: Archae - 0; Bacteria - 41; Metazoa - 198; Fungi - 63; Plants - 125; Viruses - 13; Other Eukaryotes - 1968 (source: NCBI BLink). \| chr3:4141329-4142474 REVERSE LENGTH=381 |

| | | | | |
|---|---|---|---|---|
| gBol035513 | 6.0 | 1.14E-21 | AT3G13130.1 | Symbols: \| unknown protein; FUNCTIONS IN: molecular_function unknown; INVOLVED IN: biological_process unknown; LOCATED IN: endomembrane system; EXPRESSED IN: male gametophyte; Has 140 Blast hits to 132 proteins in 41 species: Archae - 2; Bacteria - 4; Metazoa - 29; Fungi - 20; Plants - 51; Viruses - 0; Other Eukaryotes - 34 (source: NCBI BLink). \| chr3:4223008-4223613 FORWARD LENGTH=201 |
| gBol010678 | -1.9 | 4.97E-21 | | no hit |
| gBol015933 | 4.0 | 4.71E-20 | AT5G65160.1 | Symbols: TPR14 \| tetratricopeptide repeat (TPR)-containing protein \| chr5:26031457-26033668 REVERSE LENGTH=593 |
| gBol042423 | 2.0 | 5.47E-20 | AT3G24140.1 | Symbols: FMA \| basic helix-loop-helix (bHLH) DNA-binding superfamily protein \| chr3:8715525-8717772 REVERSE LENGTH=414 |
| gBol030668 | -1.5 | 1.84E-19 | AT4G02390.1 | Symbols: APP, PARP1, ATPARP1, PP \| poly(ADP-ribose) polymerase \| chr4:1050104-1053960 FORWARD LENGTH=637 |
| gBol022967 | -1.3 | 2.31E-19 | AT3G16870.1 | Symbols: GATA17 \| GATA transcription factor 17 \| chr3:5763752-5764576 REVERSE LENGTH=190 |
| gBol042544 | 1.4 | 5.83E-19 | | no hit |
| gBol010691 | -1.0 | 2.37E-18 | AT3G02080.1 | Symbols: \| Ribosomal protein S19e family protein \| chr3:364138-365161 REVERSE LENGTH=143 |
| gBol042592 | -1.7 | 4.22E-18 | AT2G14050.1 | Symbols: MCM9 \| minichromosome maintenance 9 \| chr2:5909240-5913817 FORWARD LENGTH=646 |
| gBol030644 | -1.5 | 5.15E-18 | AT4G02800.1 | Symbols: \| unknown protein; FUNCTIONS IN: molecular_function unknown; INVOLVED IN: biological_process unknown; LOCATED IN: chloroplast; EXPRESSED IN: 16 plant structures; EXPRESSED DURING: 9 growth stages; BEST Arabidopsis thaliana protein match is: unknown protein (TAIR:AT5G01970.1); Has 3209 Blast hits to 2720 proteins in 308 species: Archae - 13; Bacteria - 213; Metazoa - 1207; Fungi - 247; Plants - 183; Viruses - 21; Other Eukaryotes - 1325 (source: NCBI BLink). \| chr4:1250126-1251478 FORWARD LENGTH=333 |
| gBol010759 | 2.7 | 7.95E-18 | AT4G00670.1 | Symbols: \| Remorin family protein \| chr4:278434-279170 REVERSE LENGTH=123 |
| gBol030660 | 2.7 | 1.66E-17 | AT2G02930.1 | Symbols: ATGSTF3, GST16, GSTF3 \| glutathione S-transferase F3 \| chr2:851348-852106 REVERSE LENGTH=212 |
| gBol015854 | 1.2 | 2.30E-17 | AT5G64330.1 | Symbols: NPH3, RPT3, JK218 \| Phototropic-responsive NPH3 family protein \| chr5:25727568-25730225 FORWARD LENGTH=746 |
| gBol022897 | 2.4 | 2.88E-17 | AT3G18280.1 | Symbols: \| Bifunctional inhibitor/lipid-transfer protein/seed storage 2S albumin superfamily protein \| chr3:6267102-6267392 FORWARD LENGTH=96 |
| gBol026553 | -1.1 | 3.86E-17 | AT3G22520.1 | Symbols: \| unknown protein; INVOLVED IN: biological_process unknown; LOCATED IN: chloroplast stroma, chloroplast, chloroplast envelope; EXPRESSED IN: 24 plant structures; EXPRESSED DURING: 13 growth stages; BEST Arabidopsis thaliana protein match is: unknown protein (TAIR:AT4G14840.1); Has 717 Blast hits to 703 proteins in 179 species: Archae - 14; Bacteria - 134; Metazoa - 141; Fungi - 74; Plants - 209; Viruses - 0; Other Eukaryotes - 145 (source: NCBI BLink). \| chr3:7974984-7977406 FORWARD LENGTH=600 |
| gBol042552 | -1.2 | 4.47E-17 | AT2G05760.1 | Symbols: \| Xanthine/uracil permease family protein \| chr2:2180978-2183710 FORWARD LENGTH=520 |
| gBol022912 | 1.7 | 1.71E-16 | AT3G17820.1 | Symbols: ATGSKB6, GLN1.3, GLN1;3 \| glutamine synthetase 1.3 \| chr3:6097503-6099408 FORWARD LENGTH=354 |
| gBol010727 | -1.7 | 2.53E-16 | AT3G01710.2 | Symbols: \| TPX2 (targeting protein for Xklp2) protein family \| chr3:259952-261738 REVERSE LENGTH=388 |
| gBol015863 | -1.2 | 4.33E-16 | AT5G64420.1 | Symbols: \| DNA polymerase V family \| chr5:25756416-25761122 FORWARD LENGTH=1306 |

| | | | | |
|---|---|---|---|---|
| gBol035472 | -1.3 | 5.12E-16 | AT3G12530.1 | Symbols: PSF2 \| PSF2 \| chr3:3972604-3973864 REVERSE LENGTH=210 |
| gBol026667 | -1.5 | 6.22E-16 | AT3G20150.1 | Symbols: \| Kinesin motor family protein \| chr3:7031412-7036499 FORWARD LENGTH=1114 |
| gBol006672 | -1.6 | 6.54E-16 | AT3G49260.2 | Symbols: iqd21 \| IQ-domain 21 \| chr3:18262755-18265859 FORWARD LENGTH=471 |
| gBol022966 | -1.1 | 8.57E-16 | AT3G16950.2 | Symbols: LPD1, ptlpd1 \| lipoamide dehydrogenase 1 \| chr3:5786508-5790383 REVERSE LENGTH=623 |
| gBol042634 | 1.2 | 1.27E-15 | AT2G15970.1 | Symbols: COR413-PM1, WCOR413, WCOR413-LIKE, ATCOR413-PM1, FL3-5A3, ATCYP19 \| cold regulated 413 plasma membrane 1 \| chr2:6950163-6951012 FORWARD LENGTH=197 |
| gBol042497 | -1.1 | 1.33E-15 | AT3G25520.1 | Symbols: ATL5, PGY3, OLI5, RPL5A \| ribosomal protein L5 \| chr3:9269573-9271327 REVERSE LENGTH=301 |
| gBol010776 | 1.1 | 2.30E-15 | AT4G01150.1 | Symbols: \| unknown protein; FUNCTIONS IN: molecular_function unknown; INVOLVED IN: biological_process unknown; LOCATED IN: thylakoid, chloroplast thylakoid membrane, chloroplast, plastoglobule, chloroplast envelope; EXPRESSED IN: 23 plant structures; EXPRESSED DURING: 14 growth stages; BEST Arabidopsis thaliana protein match is: unknown protein (TAIR:AT4G38100.1); Has 323 Blast hits to 323 proteins in 59 species: Archae - 0; Bacteria - 107; Metazoa - 0; Fungi - 0; Plants - 206; Viruses - 0; Other Eukaryotes - 10 (source: NCBI BLink). \| chr4:493692-494668 FORWARD LENGTH=164 |
| gBol026662 | -1.4 | 4.91E-15 | AT3G20260.1 | Symbols: \| Protein of unknown function (DUF1666) \| chr3:7064190-7065751 REVERSE LENGTH=437 |
| gBol042478 | -1.6 | 1.56E-14 | AT3G25100.1 | Symbols: CDC45 \| cell division cycle 45 \| chr3:9144292-9146082 FORWARD LENGTH=596 |
| gBol015900 | 3.1 | 1.80E-14 | AT5G64770.1 | Symbols: RGF9 \| Encodes a root meristem growth factor (RGF). Belongs to a family of functionally redundant homologous peptides that are secreted, tyrosine-sulfated, and expressed mainly in the stem cell area and the innermost layer of central columella cells. RGFs are required for maintenance of the root stem cell niche and transit amplifying cell proliferation. Members of this family include: At5g60810 (RGF1), At1g13620 (RGF2), At2g04025 (RGF3), At3g30350 (RGF4), At5g51451 (RGF5), At4g16515 (RGF6), At3g02240 (RGF7), At2g03830 (RGF8) and At5g64770 (RGF9). \| chr5:25897681-25898085 REVERSE LENGTH=79 |
| gBol022905 | -5.2 | 3.12E-14 | AT3G18010.1 | Symbols: WOX1 \| WUSCHEL related homeobox 1 \| chr3:6161155-6163183 REVERSE LENGTH=350 |
| gBol042680 | 1.1 | 3.63E-14 | AT4G35000.1 | Symbols: APX3 \| ascorbate peroxidase 3 \| chr4:16665007-16667541 REVERSE LENGTH=287 |
| gBol042515 | 1.4 | 3.82E-14 | AT2G04570.1 | Symbols: \| GDSL-like Lipase/Acylhydrolase superfamily protein \| chr2:1594747-1596129 FORWARD LENGTH=350 |
| gBol010751 | -1.5 | 4.25E-14 | AT4G00480.1 | Symbols: ATMYC1, myc1 \| basic helix-loop-helix (bHLH) DNA-binding superfamily protein \| chr4:217106-219684 REVERSE LENGTH=526 |
| gBol026672 | -1.1 | 4.63E-14 | AT3G20050.1 | Symbols: ATTCP-1, TCP-1 \| T-complex protein 1 alpha subunit \| chr3:6998544-7002266 REVERSE LENGTH=545 |
| gBol015845 | 2.3 | 5.41E-14 | AT5G64240.2 | Symbols: AtMC3, MC3 \| metacaspase 3 \| chr5:25695836-25697249 FORWARD LENGTH=362 |
| gBol010793 | -1.1 | 5.48E-14 | AT4G00820.1 | Symbols: iqd17 \| IQ-domain 17 \| chr4:349300-351307 FORWARD LENGTH=534 |
| gBol022931 | 2.0 | 1.09E-13 | AT3G17510.1 | Symbols: CIPK1, SnRK3.16 \| CBL-interacting protein kinase 1 \| chr3:5989309-5992627 REVERSE LENGTH=444 |
| gBol035514 | -1.7 | 1.57E-13 | AT3G13160.1 | Symbols: \| Tetratricopeptide repeat (TPR)-like superfamily protein \| chr3:4229994-4231178 REVERSE LENGTH=394 |
| gBol026633 | -1.4 | 1.93E-13 | AT3G21110.2 | Symbols: PUR7, PURC \| purin 7 \| chr3:7402696-7405273 REVERSE LENGTH=411 |
| gBol042611 | 1.4 | 3.32E-13 | AT2G15220.1 | Symbols: \| Plant basic secretory protein (BSP) family protein \| chr2:6608689-6609366 FORWARD LENGTH=225 |

| | | | | |
|---|---|---|---|---|
| gBol012487 | -1.2 | 4.43E-13 | AT2G18330.1 | Symbols: \| AAA-type ATPase family protein \| chr2:7965829-7968915 FORWARD LENGTH=636 |
| gBol012498 | 5.3 | 5.69E-13 | AT2G18540.1 | Symbols: \| RmlC-like cupins superfamily protein \| chr2:8042382-8045008 REVERSE LENGTH=707 |
| gBol010772 | -1.9 | 6.31E-13 | AT4G01270.1 | Symbols: \| RING/U-box superfamily protein \| chr4:532351-534891 FORWARD LENGTH=506 |
| gBol035565 | -1.4 | 1.03E-12 | AT3G14190.1 | Symbols: \| unknown protein; BEST Arabidopsis thaliana protein match is: unknown protein (TAIR:AT5G12360.1); Has 18 Blast hits to 18 proteins in 5 species: Archae - 0; Bacteria - 0; Metazoa - 0; Fungi - 0; Plants - 18; Viruses - 0; Other Eukaryotes - 0 (source: NCBI BLink). \| chr3:4710907-4711790 FORWARD LENGTH=193 |
| gBol035478 | 1.0 | 1.19E-12 | AT3G12610.1 | Symbols: DRT100 \| Leucine-rich repeat (LRR) family protein \| chr3:4006661-4007779 REVERSE LENGTH=372 |
| gBol026636 | -1.4 | 1.55E-12 | AT3G21000.1 | Symbols: \| Gag-Pol-related retrotransposon family protein \| chr3:7363921-7365138 FORWARD LENGTH=405 |
| gBol012439 | -1.4 | 1.58E-12 | AT2G17620.1 | Symbols: CYCB2;1 \| Cyclin B2;1 \| chr2:7664164-7666261 FORWARD LENGTH=429 |
| gBol010800 | 2.0 | 1.92E-12 | AT4G01450.1 | Symbols: \| nodulin MtN21 /EamA-like transporter family protein \| chr4:608586-610487 FORWARD LENGTH=343 |
| gBol035455 | -2.0 | 4.29E-12 | AT3G12280.2 | Symbols: RBR1 \| retinoblastoma-related 1 \| chr3:3913671-3918433 REVERSE LENGTH=1012 |
| gBol026644 | -1.1 | 5.19E-12 | AT3G20670.1 | Symbols: HTA13 \| histone H2A 13 \| chr3:7229472-7229963 FORWARD LENGTH=132 |
| gBol035491 | -1.5 | 1.22E-11 | AT3G12830.1 | Symbols: \| SAUR-like auxin-responsive protein family \| chr3:4079117-4079515 REVERSE LENGTH=132 |
| gBol042630 | -2.2 | 1.64E-11 | AT2G15880.1 | Symbols: \| Leucine-rich repeat (LRR) family protein \| chr2:6918039-6920319 REVERSE LENGTH=727 |
| gBol015883 | -1.0 | 1.77E-11 | AT5G64630.2 | Symbols: FAS2, NFB01, NFB1, MUB3.9 \| Transducin/WD40 repeat-like superfamily protein \| chr5:25833298-25836158 FORWARD LENGTH=487 |
| gBol042671 | -2.3 | 4.20E-11 | AT2G16660.1 | Symbols: \| Major facilitator superfamily protein \| chr2:7218930-7221592 REVERSE LENGTH=546 |
| gBol030682 | -1.3 | 6.61E-11 | AT4G02110.1 | Symbols: \| transcription coactivators \| chr4:935191-940191 FORWARD LENGTH=1329 |
| gBol042537 | 1.3 | 7.60E-11 | AT2G05100.1 | Symbols: LHCB2.1, LHCB2 \| photosystem II light harvesting complex gene 2.1 \| chr2:1823449-1824331 REVERSE LENGTH=265 |
| gBol042501 | 1.2 | 7.73E-11 | AT2G04350.2 | Symbols: LACS8 \| AMP-dependent synthetase and ligase family protein \| chr2:1516086-1519178 FORWARD LENGTH=720 |
| gBol041311 | 2.4 | 1.26E-10 | AT3G44990.1 | Symbols: XTR8, ATXTR8, XTH31 \| xyloglucan endo-transglycosylase-related 8 \| chr3:16447280-16448678 REVERSE LENGTH=293 |
| gBol035454 | -1.9 | 2.04E-10 | AT3G12270.1 | Symbols: ATPRMT3, PRMT3 \| protein arginine methyltransferase 3 \| chr3:3910642-3913122 FORWARD LENGTH=601 |
| gBol010683 | -3.0 | 4.38E-10 | AT3G02150.2 | Symbols: PTF1, TFPD \| plastid transcription factor 1 \| chr3:391522-392589 FORWARD LENGTH=355 |
| gBol035546 | -2.7 | 6.46E-10 | AT3G13690.1 | Symbols: \| Protein kinase protein with adenine nucleotide alpha hydrolases-like domain \| chr3:4486920-4490011 FORWARD LENGTH=753 |
| gBol006655 | -1.6 | 8.49E-10 | AT3G49600.1 | Symbols: UBP26, SUP32, ATUBP26 \| ubiquitin-specific protease 26 \| chr3:18380942-18386662 REVERSE LENGTH=1067 |
| gBol022914 | 1.4 | 1.15E-09 | AT3G17800.1 | Symbols: \| Protein of unknown function (DUF760) \| chr3:6091248-6092873 REVERSE LENGTH=421 |
| gBol022883 | -1.0 | 1.81E-09 | AT3G18600.1 | Symbols: \| P-loop containing nucleoside triphosphate hydrolases superfamily protein \| chr3:6399724-6403007 REVERSE LENGTH=568 |

| | | | | |
|---|---|---|---|---|
| gBol022979 | 1.1 | 2.50E-09 | AT3G16570.1 | Symbols: RALF23, ATRALF23 \| rapid alkalinization factor 23 \| chr3:5644748-5645164 FORWARD LENGTH=138 |
| gBol026576 | -1.2 | 3.03E-09 | AT4G15000.1 | Symbols: \| Ribosomal L27e protein family \| chr4:8571896-8572303 FORWARD LENGTH=135 |
| gBol010666 | 3.2 | 3.07E-09 | AT3G02500.1 | Symbols: \| unknown protein; BEST Arabidopsis thaliana protein match is: unknown protein (TAIR:AT5G16030.1); Has 49 Blast hits to 49 proteins in 10 species: Archae - 0; Bacteria - 0; Metazoa - 0; Fungi - 0; Plants - 49; Viruses - 0; Other Eukaryotes - 0 (source: NCBI BLink). \| chr3:519364-521628 FORWARD LENGTH=293 |
| gBol012502 | 1.8 | 3.59E-09 | AT2G18690.1 | Symbols: \| unknown protein; FUNCTIONS IN: molecular_function unknown; INVOLVED IN: biological_process unknown; LOCATED IN: membrane; EXPRESSED IN: 17 plant structures; EXPRESSED DURING: 9 growth stages; CONTAINS InterPro DOMAIN/s: Protein of unknown function DUF975 (InterPro:IPR010380); BEST Arabidopsis thaliana protein match is: unknown protein (TAIR:AT2G18680.1); Has 213 Blast hits to 211 proteins in 20 species: Archae - 0; Bacteria - 8; Metazoa - 0; Fungi - 0; Plants - 205; Viruses - 0; Other Eukaryotes - 0 (source: NCBI BLink). \| chr2:8097650-8098618 FORWARD LENGTH=322 |
| gBol035509 | 1.4 | 4.56E-09 | AT3G13080.1 | Symbols: ATMRP3, MRP3, ABCC3 \| multidrug resistance-associated protein 3 \| chr3:4196019-4201250 REVERSE LENGTH=1514 |
| gBol015936 | 1.4 | 5.77E-09 | AT5G65170.1 | Symbols: \| VQ motif-containing protein \| chr5:26041218-26042306 FORWARD LENGTH=362 |
| gBol010967 | -1.0 | 6.37E-09 | AT3G10480.1 | Symbols: ANAC050, NAC050 \| NAC domain containing protein 50 \| chr3:3264410-3266781 FORWARD LENGTH=447 |
| gBol010734 | -1.3 | 9.88E-09 | AT3G01800.1 | Symbols: \| Ribosome recycling factor \| chr3:286020-287543 FORWARD LENGTH=267 |
| gBol022898 | 1.3 | 1.42E-08 | AT3G18260.1 | Symbols: \| Reticulon family protein \| chr3:6260328-6261504 REVERSE LENGTH=225 |
| gBol035467 | -4.5 | 1.48E-08 | AT3G12500.1 | Symbols: ATHCHIB, PR3, PR-3, CHI-B, B-CHI, HCHIB \| basic chitinase \| chr3:3962501-3963984 REVERSE LENGTH=335 |
| gBol026545 | -1.3 | 1.50E-08 | AT3G22660.1 | Symbols: \| rRNA processing protein-related \| chr3:8016237-8017118 REVERSE LENGTH=293 |
| gBol042455 | -1.8 | 2.95E-08 | AT3G54670.3 | Symbols: TTN8 \| Structural maintenance of chromosomes (SMC) family protein \| chr3:20235818-20243701 FORWARD LENGTH=1239 |
| gBol010697 | 2.7 | 4.41E-08 | AT1G79680.1 | Symbols: WAKL10, ATWAKL10 \| WALL ASSOCIATED KINASE (WAK)-LIKE 10 \| chr1:29980188-29982749 REVERSE LENGTH=769 |
| gBol026592 | 1.2 | 5.07E-08 | AT3G21760.1 | Symbols: HYR1 \| UDP-Glycosyltransferase superfamily protein \| chr3:7667099-7668556 FORWARD LENGTH=485 |
| gBol042574 | 1.5 | 6.63E-08 | AT2G13360.2 | Symbols: AGT, AGT1, SGAT \| alanine:glyoxylate aminotransferase \| chr2:5539417-5540902 REVERSE LENGTH=401 |
| gBol042602 | 1.4 | 6.93E-08 | AT2G14750.1 | Symbols: APK, AKN1, ATAKN1, APK1 \| APS kinase \| chr2:6314128-6315501 FORWARD LENGTH=276 |
| gBol042543 | 2.2 | 7.80E-08 | | no hit |
| gBol026593 | 1.6 | 9.17E-08 | AT3G21760.1 | Symbols: HYR1 \| UDP-Glycosyltransferase superfamily protein \| chr3:7667099-7668556 FORWARD LENGTH=485 |
| gBol012444 | 1.1 | 1.01E-07 | AT2G17695.3 | Symbols: \| FUNCTIONS IN: molecular_function unknown; INVOLVED IN: biological_process unknown; CONTAINS InterPro DOMAIN/s: Domain of unknown function DUF1990 (InterPro:IPR018960). \| chr2:7684214-7685009 REVERSE LENGTH=205 |
| gBol010742 | 1.4 | 1.27E-07 | AT4G00050.1 | Symbols: UNE10 \| basic helix-loop-helix (bHLH) DNA-binding superfamily protein \| chr4:17863-19848 FORWARD LENGTH=399 |

| | | | | |
|---|---|---|---|---|
| gBol023016 | 1.3 | 1.76E-07 | AT3G15810.1 | Symbols: \| Protein of unknown function (DUF567) \| chr3:5348054-5349178 REVERSE LENGTH=220 |
| gBol022970 | 1.8 | 2.25E-07 | AT3G16857.1 | Symbols: ARR1, RR1 \| response regulator 1 \| chr3:5756113-5758853 FORWARD LENGTH=669 |
| gBol026656 | -1.1 | 2.77E-07 | AT3G20390.1 | Symbols: \| endoribonuclease L-PSP family protein \| chr3:7110227-7111695 REVERSE LENGTH=187 |
| gBol042548 | 1.5 | 4.90E-07 | AT2G05620.1 | Symbols: PGR5 \| proton gradient regulation 5 \| chr2:2081204-2081687 REVERSE LENGTH=133 |
| gBol010720 | -1.3 | 6.36E-07 | AT3G01600.1 | Symbols: anac044, NAC044 \| NAC domain containing protein 44 \| chr3:229365-231105 FORWARD LENGTH=370 |
| gBol022884 | -2.8 | 6.56E-07 | AT3G18590.1 | Symbols: ENODL5, AtENODL5 \| early nodulin-like protein 5 \| chr3:6398670-6399337 FORWARD LENGTH=188 |
| gBol030652 | -1.2 | 7.91E-07 | AT4G02660.1 | Symbols: \| Beige/BEACH domain ;WD domain, G-beta repeat protein \| chr4:1159927-1173791 REVERSE LENGTH=3527 |
| gBol042468 | 1.1 | 8.71E-07 | AT3G24730.1 | Symbols: \| mRNA splicing factor, thioredoxin-like U5 snRNP \| chr3:9030152-9030894 REVERSE LENGTH=159 |
| gBol010949 | 2.2 | 1.18E-06 | AT3G10815.1 | Symbols: \| RING/U-box superfamily protein \| chr3:3385009-3385608 REVERSE LENGTH=199 |
| gBol030661 | 2.9 | 3.23E-06 | AT4G02520.1 | Symbols: ATGSTF2, ATPM24.1, ATPM24, GST2, GSTF2 \| glutathione S-transferase PHI 2 \| chr4:1110673-1111531 REVERSE LENGTH=212 |
| gBol041291 | 1.0 | 5.74E-06 | AT3G44300.1 | Symbols: NIT2, AtNIT2 \| nitrilase 2 \| chr3:15983351-15985172 FORWARD LENGTH=339 |
| gBol042412 | 2.5 | 6.31E-06 | AT3G23810.1 | Symbols: SAHH2, ATSAHH2 \| S-adenosyl-l-homocysteine (SAH) hydrolase 2 \| chr3:8588013-8589671 REVERSE LENGTH=485 |
| gBol022866 | -2.9 | 8.19E-06 | AT5G48485.1 | Symbols: DIR1 \| Bifunctional inhibitor/lipid-transfer protein/seed storage 2S albumin superfamily protein \| chr5:19646317-19646625 REVERSE LENGTH=102 |
| gBol010797 | 1.2 | 9.42E-06 | AT4G00780.1 | Symbols: \| TRAF-like family protein \| chr4:334779-336120 FORWARD LENGTH=299 |
| gBol026599 | 1.6 | 1.04E-05 | AT3G21700.3 | Symbols: SGP2 \| Ras-related small GTP-binding family protein \| chr3:7644581-7646190 FORWARD LENGTH=292 |
| gBol023009 | -1.2 | 1.27E-05 | AT3G15880.3 | Symbols: WSIP2 \| WUS-interacting protein 2 \| chr3:5364792-5371869 REVERSE LENGTH=1125 |
| gBol026623 | 1.3 | 1.34E-05 | AT3G21230.1 | Symbols: 4CL5 \| 4-coumarate:CoA ligase 5 \| chr3:7448231-7451947 REVERSE LENGTH=570 |
| gBol042581 | -1.4 | 1.54E-05 | AT2G13600.1 | Symbols: \| Pentatricopeptide repeat (PPR) superfamily protein \| chr2:5671493-5673586 FORWARD LENGTH=697 |
| gBol026609 | -1.1 | 1.60E-05 | AT4G15560.1 | Symbols: CLA1, DEF, CLA, DXS, DXPS2 \| Deoxyxylulose-5-phosphate synthase \| chr4:8884218-8887254 FORWARD LENGTH=717 |
| gBol006669 | -1.3 | 2.67E-05 | AT3G49320.1 | Symbols: \| Metal-dependent protein hydrolase \| chr3:18288381-18290629 REVERSE LENGTH=354 |
| gBol042499 | -2.0 | 3.39E-05 | AT2G04160.1 | Symbols: AIR3 \| Subtilisin-like serine endopeptidase family protein \| chr2:1401450-1407694 REVERSE LENGTH=772 |
| gBol035498 | 1.3 | 3.58E-05 | AT3G12977.1 | Symbols: \| NAC (No Apical Meristem) domain transcriptional regulator superfamily protein \| chr3:4143832-4145860 FORWARD LENGTH=279 |
| gBol010692 | -2.9 | 3.62E-05 | AT3G01015.1 | Symbols: \| TPX2 (targeting protein for Xklp2) protein family \| chr3:1798-4017 REVERSE LENGTH=488 |
| gBol022925 | 2.2 | 3.65E-05 | AT3G17609.2 | Symbols: HYH \| HY5-homolog \| chr3:6023971-6024585 FORWARD LENGTH=149 |
| gBol042457 | 1.3 | 4.37E-05 | AT3G24520.1 | Symbols: AT-HSFC1, HSFC1 \| heat shock transcription factor C1 \| chr3:8941455-8942531 FORWARD LENGTH=330 |
| gBol012506 | -1.5 | 5.09E-05 | AT4G30200.1 | Symbols: VEL1, VIL2 \| vernalization5/VIN3-like \| chr4:14786852-14789103 REVERSE LENGTH=685 |

| | | | | |
|---|---|---|---|---|
| gBol010802 | 1.5 | 6.33E-05 | AT4G01450.1 | Symbols: \| nodulin MtN21 /EamA-like transporter family protein \| chr4:608586-610487 FORWARD LENGTH=343 |
| gBol042541 | -3.5 | 7.01E-05 | AT3G11680.1 | Symbols: \| Aluminium activated malate transporter family protein \| chr3:3686995-3689329 REVERSE LENGTH=488 |
| gBol026629 | 1.5 | 9.83E-05 | AT3G21150.1 | Symbols: BBX32 \| B-box 32 \| chr3:7412713-7413390 REVERSE LENGTH=225 |
| gBol042494 | -1.1 | 0.00010 | AT4G12790.5 | Symbols: \| P-loop containing nucleoside triphosphate hydrolases superfamily protein \| chr4:7517280-7518892 REVERSE LENGTH=271 |
| gBol035471 | 1.7 | 0.00011 | AT3G12520.1 | Symbols: SULTR4;2 \| sulfate transporter 4;2 \| chr3:3967976-3971891 REVERSE LENGTH=677 |
| gBol035458 | 2.1 | 0.00015 | AT3G12320.1 | Symbols: \| unknown protein; BEST Arabidopsis thaliana protein match is: unknown protein (TAIR:AT5G06980.4); Has 102 Blast hits to 102 proteins in 16 species: Archae - 0; Bacteria - 0; Metazoa - 0; Fungi - 0; Plants - 98; Viruses - 0; Other Eukaryotes - 4 (source: NCBI BLink). \| chr3:3924034-3925262 FORWARD LENGTH=269 |
| gBol042679 | 1.4 | 0.00063 | AT2G16720.1 | Symbols: MYB7, ATMYB7, ATY49 \| myb domain protein 7 \| chr2:7255669-7256550 REVERSE LENGTH=269 |
| gBol026596 | 2.8 | 0.00085 | AT3G21720.1 | Symbols: ICL \| isocitrate lyase \| chr3:7652789-7655873 REVERSE LENGTH=576 |
| gBol022962 | 1.8 | 0.00144 | AT3G17020.1 | Symbols: \| Adenine nucleotide alpha hydrolases-like superfamily protein \| chr3:5802728-5804063 REVERSE LENGTH=163 |
| gBol042613 | 1.2 | 0.00147 | AT4G23690.1 | Symbols: \| Disease resistance-responsive (dirigent-like protein) family protein \| chr4:12339152-12339715 REVERSE LENGTH=187 |
| gBol010928 | 1.6 | 0.00156 | AT3G11420.1 | Symbols: \| Protein of unknown function (DUF604) \| chr3:3591834-3594323 FORWARD LENGTH=505 |
| gBol042405 | 1.0 | 0.00162 | AT3G23410.1 | Symbols: ATFAO3, FAO3 \| fatty alcohol oxidase 3 \| chr3:8382860-8386024 FORWARD LENGTH=746 |
| gBol015888 | -2.2 | 0.00163 | | no hit |
| gBol035462 | -1.7 | 0.00184 | AT3G12410.1 | Symbols: \| Polynucleotidyl transferase, ribonuclease H-like superfamily protein \| chr3:3946267-3946959 REVERSE LENGTH=230 |
| gBol015881 | 1.0 | 0.00208 | AT5G64620.1 | Symbols: C/VIF2, ATC/VIF2 \| cell wall / vacuolar inhibitor of fructosidase 2 \| chr5:25831875-25832417 FORWARD LENGTH=180 |
| gBol042631 | -1.6 | 0.00224 | AT2G15890.1 | Symbols: MEE14 \| maternal effect embryo arrest 14 \| chr2:6921196-6921978 REVERSE LENGTH=203 |
| gBol035495 | 1.7 | 0.00280 | AT3G12910.1 | Symbols: \| NAC (No Apical Meristem) domain transcriptional regulator superfamily protein \| chr3:4109417-4110648 FORWARD LENGTH=303 |
| gBol042586 | 2.7 | 0.00408 | AT2G13810.1 | Symbols: ALD1 \| AGD2-like defense response protein 1 \| chr2:5768489-5772138 FORWARD LENGTH=456 |
| gBol035474 | 1.5 | 0.00439 | AT3G12580.1 | Symbols: HSP70, ATHSP70 \| heat shock protein 70 \| chr3:3991487-3993689 REVERSE LENGTH=650 |
| gBol022893 | 2.6 | 0.00442 | AT3G18400.1 | Symbols: anac058, NAC058 \| NAC domain containing protein 58 \| chr3:6318745-6320593 REVERSE LENGTH=314 |
| gBol022871 | 1.2 | 0.00460 | AT3G18773.1 | Symbols: \| RING/U-box superfamily protein \| chr3:6466304-6466966 FORWARD LENGTH=220 |
| gBol012489 | 2.8 | 0.00466 | AT2G18360.1 | Symbols: \| alpha/beta-Hydrolases superfamily protein \| chr2:7976848-7979221 REVERSE LENGTH=313 |
| gBol010760 | 1.7 | 0.00470 | AT4G00700.1 | Symbols: \| C2 calcium/lipid-binding plant phosphoribosyltransferase family protein \| chr4:286260-289369 FORWARD LENGTH=1006 |
| gBol012456 | -1.5 | 0.00483 | AT2G17840.1 | Symbols: ERD7 \| Senescence/dehydration-associated protein-related \| chr2:7755923-7757798 REVERSE LENGTH=452 |
