# Supplementary material for: Low Temperature Affects Stem Cell Maintenance in Brassica oleracea Seedlings
Source: Front Plant Sci. 2016 Jun 8;7:800. doi: 10.3389/fpls.2016.00800 (PMC4896912; doi:10.3389/fpls.2016.00800)
Supplement: Supplementary file 5 [file Table_5.PDF]

Supplemental material to:
de Jonge J, Kodde J, Severing EI, Bonnema G, Angenent GC, Immink RGH and Groot SPC (2016) Low Temperature Affects Stem Cell Maintenance in Brassica oleracea Seedlings. Front. Plant Sci. 7:800. doi: 10.3389/fpls.2016.00800

Supplemental Table S5. Top 20 over-represented Gene Ontology (GO) terms (p-value < 0.05 after Benjamini Hochberg correction) in selected differentially expressed gene sets based on comparison of non-induced and cold-induced plants at two days after the start of the experiment. For each comparison, indicated by the sub-headings, only the top 20 over-represented GO terms is given. GO-ID is the Gene Ontology identifier. The description of the over-represented GO-IDs is given in the last column. The values in column 'N' and 'Background' correspond to the number of genes with that particular GO-ID term annotation in the differentially expressed gene set and a control background set, respectively.
Day2

GO-ID	P-value	N	Background	Description
15979	2,40E-25	151	539	photosynthesis
19684	4,19E-24	129	431	photosynthesis, light reaction
10207	2,78E-17	81	237	photosystem II assembly
33205	3,55E-14	99	363	cell cycle cytokinesis
911	3,55E-14	98	358	cytokinesis by cell plate formation
910	1,85E-11	100	402	cytokinesis
22402	4,75E-11	168	863	cell cycle process
51301	4,75E-11	124	562	cell division
6364	5,82E-11	102	423	rRNA processing
16072	6,23E-11	102	424	rRNA metabolic process
6996	1,12E-10	473	3314	organelle organization
7049	1,49E-10	193	1059	cell cycle
10114	1,66E-10	59	182	response to red light
9892	6,62E-10	178	965	negative regulation of metabolic process
9773	1,08E-09	37	84	photosynthetic electron transport in photosystem I
10218	1,45E-09	57	180	response to far red light
19222	1,70E-09	598	4467	regulation of metabolic process
9767	6,13E-09	41	106	photosynthetic electron transport chain
9743	8,73E-09	228	1375	response to carbohydrate stimulus
34470	1,81E-08	107	497	ncRNA processing
