# Supplementary material for: Low Temperature Affects Stem Cell Maintenance in Brassica oleracea Seedlings
Source: Front Plant Sci. 2016 Jun 8;7:800. doi: 10.3389/fpls.2016.00800 (PMC4896912; doi:10.3389/fpls.2016.00800)
Supplement: Supplementary file 6 [file Table_6.PDF]

Supplemental material to:
de Jonge J, Kodde J, Severing EI, Bonnema G, Angenent GC, Immink RGH and Groot SPC (2016) Low Temperature Affects Stem Cell Maintenance in Brassica oleracea Seedlings. Front. Plant Sci. 7:800. doi: 10.3389/fpls.2016.00800

Supplemental Table S6. Top 20 over-represented Gene Ontology (GO) terms (p-value < 0.05 after Benjamini Hochberg correction) in selected differentially expressed gene sets based on comparison of non-induced and cold-induced plants at two and seven days after the start of the experiment. For each comparison, indicated by the sub-headings, only the top 20 over-represented GO terms is given. GO-ID is the Gene Ontology identifier. The description of the over-represented GO-IDs is given in the last column. The values in column 'N' and 'Background' correspond to the number of genes with that particular GO-ID term annotation in the differentially expressed gene set and a control background set, respectively.
Day7

GO-ID	P-value	N	Background	Description
8283	1,01E-50	170	287	cell proliferation
6260	4,79E-43	228	496	DNA replication
34968	2,23E-40	196	408	histone lysine methylation
32259	1,27E-39	349	952	methylation
43414	2,33E-39	345	940	macromolecule methylation
6730	8,37E-38	354	989	one-carbon metabolic process
6275	1,06E-35	132	237	regulation of DNA replication
51567	1,09E-35	154	302	histone H3-K9 methylation
6261	4,53E-33	183	408	DNA-dependent DNA replication
16570	4,53E-33	241	609	histone modification
6325	1,33E-31	320	919	chromatin organization
16568	2,99E-29	269	745	chromatin modification
16569	3,82E-29	256	697	covalent chromatin modification
16571	1,43E-28	199	493	histone methylation
51052	1,43E-28	150	326	regulation of DNA metabolic process
8213	1,83E-28	202	505	protein amino acid alkylation
6479	1,83E-28	202	505	protein amino acid methylation
51276	1,97E-28	368	1148	chromosome organization
911	1,37E-27	159	362	cytokinesis by cell plate formation
33205	2,41E-27	160	367	cell cycle cytokinesis
