# Supplementary material for: Low Temperature Affects Stem Cell Maintenance in Brassica oleracea Seedlings
Source: Front Plant Sci. 2016 Jun 8;7:800. doi: 10.3389/fpls.2016.00800 (PMC4896912; doi:10.3389/fpls.2016.00800)
Supplement: Supplementary file 7 [file Table_7.PDF]

Supplemental material to:
de Jonge J, Kodde J, Severing EI, Bonnema G, Angenent GC, Immink RGH and Groot SPC (2016) Low Temperature Affects Stem Cell Maintenance in Brassica oleracea Seedlings. Front. Plant Sci. 7:800. doi: 10.3389/fpls.2016.00800

Table S7. Cold affects a higher number of genes expressed in the meristematic region of the SAM. All comparisons are made towards seedlings of the same ages without cold treatment.

	Genes specifically expressed in the centre (meristematic) of the SAM (S4 area in Yadav et al, 2009)				Genes specifically expressed in the flanking region (differentiation) of the SAM (S7 area in Yadav et al, 2009)			
**Treatment**	**Number of significantly differentially expressed genes**	**Percentage of significantly differentially expressed genes**	**Percentage up-regulated**	**Percentage down-regulated**	**Number of significantly differentially expressed genes**	**Percentage of significantly differentially expressed genes**	**Percentage up-regulated**	**Percentage down-regulated**
Cold treatment in a sensitive seed lot, Day 2	27	14	52	48	132	13	79	21
Cold treatment in a resistant seed lot, Day 2	11	5	55	45	61	6	64	36
Blind seedlings of a sensitive cold induced line, Day 7	91	41	59	41	469	40	46	54
Normal seedlings of a sensitive cold induced line, Day 7	23	11	87	13	106	10	72	28
