# Supplementary material for: Low Temperature Affects Stem Cell Maintenance in Brassica oleracea Seedlings
Source: Front Plant Sci. 2016 Jun 8;7:800. doi: 10.3389/fpls.2016.00800 (PMC4896912; doi:10.3389/fpls.2016.00800)
Supplement: Supplementary file 9 [file Image_2.PDF]

Supplemental material to:

de Jonge J, Kodde J, Severing EI, Bonnema G, Angenent GC, Immink RGH and Groot SPC (2016) Low Temperature Affects Stem Cell Maintenance in Brassica oleracea Seedlings. Front. Plant Sci. 7:800. doi: 10.3389/fpls.2016.00800

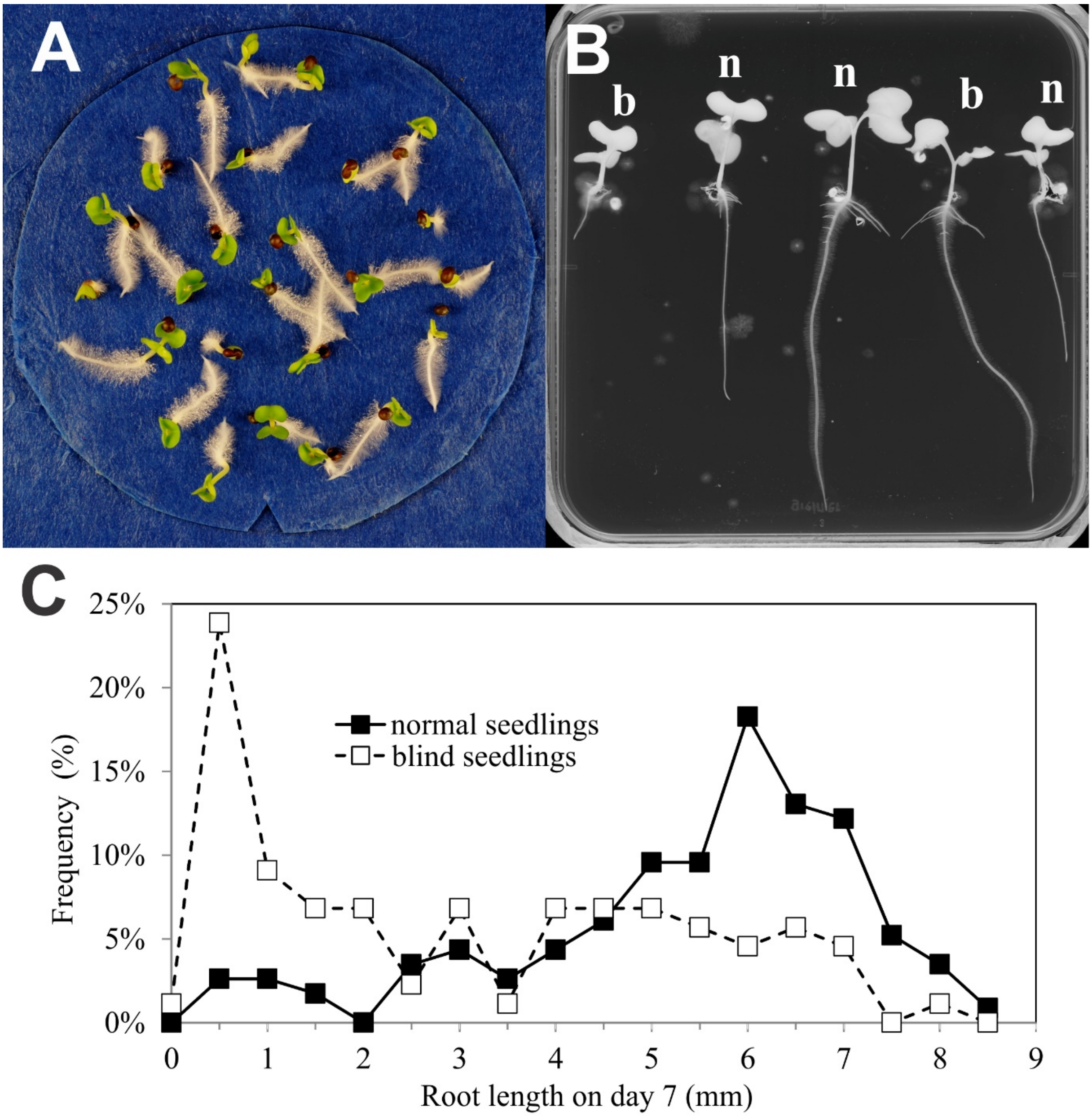

Figure S2. Root development of AG 5010 *B. oleracea* seedlings after cold induction. The normal or blind seedling identity was determined ten days after transfer from the cold to 20 °C. (A) Variation in seedling root morphologies four days after transfer from the cold. (B) variation in seedling root growth ten days after transfer (n = normal, b = blind). (C) Frequency distribution of root length ten days after transfer for seedlings with (n = normal) or without (b = blind) shoot development
