# Supplementary material for: Low Temperature Affects Stem Cell Maintenance in Brassica oleracea Seedlings
Source: Front Plant Sci. 2016 Jun 8;7:800. doi: 10.3389/fpls.2016.00800 (PMC4896912; doi:10.3389/fpls.2016.00800)
Supplement: Supplementary file 10 [file Image_3.PDF]

Supplemental material to:

de Jonge J, Kodde J, Severing EI, Bonnema G, Angenent GC, Immink RGH and Groot SPC (2016) Low Temperature Affects Stem Cell Maintenance in Brassica oleracea Seedlings. Front. Plant Sci. 7:800. doi: 10.3389/fpls.2016.00800

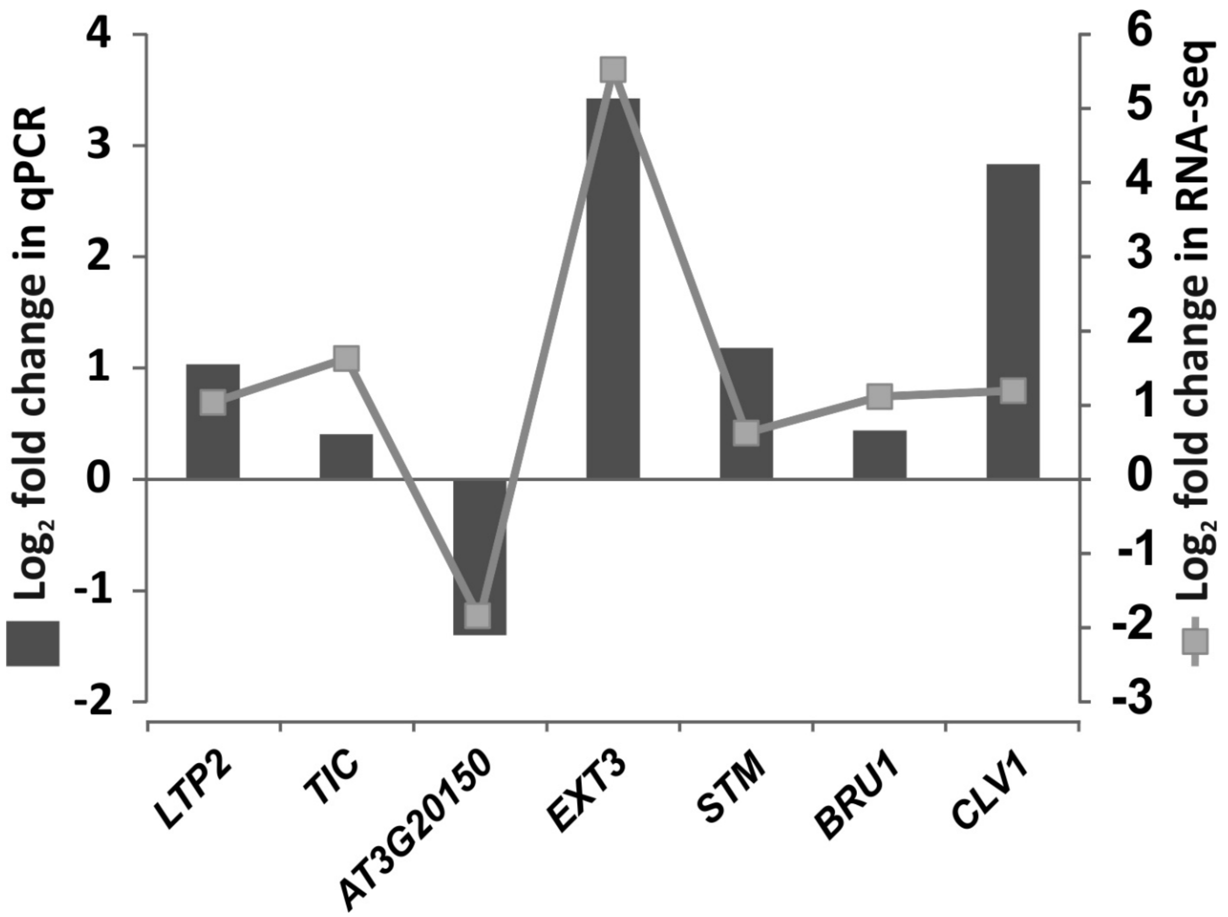

Figure S3. Validation of the RNA-seq data by qPCR for six genes. The bars represent the log^2 of relative expression ratio, while the small squares connected with a line represents the log^2 of the RNA-seq expression in estimation of fragments per kilobase of exon per million fragments mapped (FPKM).
